# Computational discovery of potential therapeutic agents against brain-eating amoeba (*Naegleria fowleri*)

**DOI:** 10.1371/journal.pone.0327621

**Published:** 2025-07-11

**Authors:** Jacopo Zattoni, Richard F. Ludueña, Maral Aminpour, Jack A. Tuszynski

**Affiliations:** 1 Department of Biomedical Engineering, University of Alberta, Donadeo Innovation Centre for Engineering, Edmonton, Alberta, Canada; 2 University of Texas Health Sciences Center, San Antonio, Texas, United States of America; 3 DIMEAS, Politecnico di Torino, Torino, Piedmont, Italy; 4 Department of Physics, University of Alberta, Edmonton, Alberta, Canada; 5 Department of Data Science and Engineering, The Silesian University of Technology, Gliwice, Poland; Iran University of Medical Sciences, IRAN, ISLAMIC REPUBLIC OF

## Abstract

*Naegleria fowleri* is a human and animal pathogen well-known for its ability to digest neurons and astrocytes of the host’s brain, causing a haemorrhagic and necrotizing inflammation called *Primary Amoebic Meningoencephalitis*. Although infections are rare, the mortality rate is over 97%, due to both the non-specificity of the symptoms and the absence of an effective treatment. In this work we employed bioinformatics tools to evaluate the possibility of treating the infection with tubulin-targeting compounds, which we regard as the most promising approach given the unclear view on the pathogenic factors in *N. fowleri*, the divergence of the amoeba’s tubulins from the human counterparts, and how well-established microtubule-targeting therapies are in clinical practices. The amoeba’s tubulin sequences were analyzed and compared to the human tubulins to conjecture the role of their differences in drugs resistance. The binding affinity of the compounds was computed for both species by performing docking simulations using Chemical Computing Group’s MOE and CCSB’s AutoDock4 and AutoDock Vina. The results were analyzed using a consensus method to increase their reliability. We found that the amoeba’s mitotic tubulins show a significant number of changes that are expected to decrease their affinity for tubulin-targeting compounds. We identified the Colchicine binding site as the most suitable target, and propose that Colchicine analogs retain their ability to bind to the amoeba’s tubulins *in vivo.* The selectivity of the compounds for the pathogen however remains an issue. The changes in the amino. acid sequences in the Colchicine site could create a template for designing novel derivatives with an improved selectivity for the parasite and a safer profile for the patient. We therefore believe that our results could be the starting point for a rational derivatization of the selected ligands, leading to the development of an effective treatment for *Naegleria fowleri* infection.

## 1. Introduction

*Naegleria fowleri* is a thermophilic, free-living amoeba belonging to the genus Naegleria. First discovered in Australia in 1965, *N. fowleri* lives on every continent except Antarctica [1], and is a rare but extremely fatal human pathogen.

This amoeba exhibits three distinct phenotypic forms: the trophozoite, responsible for reproduction and infection; the flagellate or natant form, emerging in nutrient-poor conditions; and the cyst or dormant form. Neither of the latter two forms are infective or reproductive, nor have been found in infected patients [1–3].

*N. fowleri* is commonly known as the brain-eating amoeba: by infecting the central nervous system (CNS) of animals and humans, it digests the host’s neurons and astrocytes through a process called *trogocytosis* — essentially, cellular nibbling [1,3].

The disruption of the host’s nervous cells results in a condition called Primary Amoebic Meningoencephalitis (PAM), characterized by haemorrhagic and necrotic inflammation of brain tissues, rapidly progressing to cerebral edema and potentially cerebellar herniation [4]. Frequently, PAM also causes focal demyelination in the white matter and spinal cord of the affected individuals [1,5,6].

*N. fowleri* preferentially lives in warm, aqueous environments [7–13], but its presence has been documented in soil and air as well [2,14–19]. The infection happens when contaminated water enters the nares [2,13], from where the amoeba can reach the frontal lobe of the host’s brain traveling along the nasal neuroepithelium. Once in the brain, *N. fowleri* replicates through closed binary fission, and spreads to the back of the brain and the cerebrospinal fluid (CSF), causing damage to the hypothalamus, the brainstem and the midbrain along the way [1,5,20].

The differential diagnosis of PAM in infected patients presents a significant challenge, even if using lab analysis [21]. This is due to the fast evolution of the pathology, and the non-specific symptoms related to PAM [1,3,7,22]. The incubation period may last between one and 16 days, and death generally occurs within 14 days from the symptoms’ onset. Initially, PAM resembles flu, such that many patients do not seek medical assistance until their condition deteriorates significantly. As the infection spreads throughout the brain, symptoms evolve into seizures and coma, which could be attributed to a wide range of other CNS pathologies and conditions.

*N. fowleri* infection is generally fatal, with a mortality rate above 97% [23,24]. However, its incidence is rare: between 1962 and 2023, only 488 cases have been documented worldwide, with 11 survivors [23]. Although the infections have been registered mainly in the USA, Europe and Australia, PAM’s incidence is probably underestimated, especially in developing countries. Indeed, 146 cases were registered only in the region of Karachi, Pakistan, from year 2008–2019 [25], with recent reports of infections in other parts of Asia as well. [7,10] In these regions, the absence of awareness and control measures, coupled with poor health care infrastructure, may cause this infection to go unnoticed among a wide variety of other infections. Interestingly, several studies [2,7,10,25] have revealed that PAM has a marked seasonality: cases are more frequent during summer, mainly because of the increased use of water for recreational activities, one of the main vectors for the infection. This suggests that the influence of global warming on temperature and precipitations could favor the proliferation of the amoeba, and increase its incidence worldwide in the near future [2,7,11,25–27].

**Fig 1:**
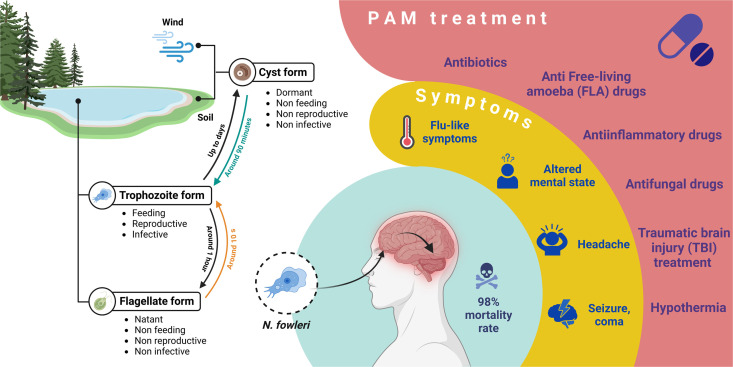
Infographic illustrating the life cycle, infection pathway, symptoms, and treatment options for the brain-eating amoeba (*N. fowleri).* The life cycle begins with the cyst form, which is dormant and can survive for years outside of water. When conditions are favorable, cysts transform into trophozoites—the feeding, reproductive and infective form, which can in turn convert to the flagellate in absence of food. Trophozoites are responsible for animal and human infection. After reaching the frontal lobe of the host via nasal passage, they digest neurons and astrocytes causing a condition called Primary Amoebic Meningoencephalitis (PAM). Symptoms at the onset are flu-like, including headache, fever and altered mental state, and evolving within two weeks into seizures and coma. The current state-of-the-art treatment is a combination of drugs (antibiotics, anti-FLA drugs, antifungal and anti-inflammatory drugs), along with physical therapies commonly used to treat brain injuries. Nonetheless, the survival rate is estimated to be around 3%.

*N. fowleri* can be eliminated by either treating water with chlorine or boiling it prior to its use, when possible [28–30]. Upon infection, the treatment for PAM involves a combination of drugs targeting various aspects of the disease: the antifungals Fluconazole [31,32] and Amphotericin B [32,33]; the anti-inflammatory Dexamethasone [34]; the antibiotics Rifampin [32,35] and Azithromycin [36,37]; and the anti-free-living amoeba (FLA), antimicrobial, anti-leishmania and anticancer Miltefosine [38,39]. Amphotericin B and Azithromycin are considered the cornerstone of the treatment thanks to their synergistic effect [36], while the other drugs used provide broad-spectrum coverage [40]. Additionally, the application of traumatic brain injury principles has been shown to be promising for a positive outcome [41]. The most commonly used drugs and their mode of action are detailed in Table S1 of the Supporting information. An overview of the treatment can be found in Fig 1.

Current treatments for PAM have serious limitations. The need to cross the blood-brain barrier (BBB) often requires high doses to achieve a minimum inhibitory concentration (MIC) in the brain, resulting in marked toxicity, and some of the compounds currently in use already exhibit important side effects at low doses. Furthermore, high doses of anti-inflammatories may lead to the inhibition of the immune response, and, with the only exception of miltefosine, none of the drugs clinically used is specific for *N. fowleri*, prompting the identification of novel therapeutic strategies.

One of the main reasons why this infection is so difficult to cure is the incomplete knowledge we have on the mechanisms responsible for *N. fowleri*’s pathogenicity. Genomic comparison to the harmless relatives *N. gruberi, N. lovaniensis, and N. pringsheimi* showed that differential gene expression in *N. fowleri* is as relevant to its pathogenesis as the presence of unique proteins and cellular actors [42,43].

*N. fowleri* displays also several mechanisms to protect itself from external damages. A heat-shock protein (HSP70) identified in the cytoplasm, the pseudopodia and the amoebastomes, provides protection from temperature-induced damages [44]. Additionally, a CD-59-like membrane protein protects the amoeba from complement-mediated cell lysis [42]. Studies have also shown *N. fowleri*’s ability to delay the host’s immune system recognition, by internalizing the antibodies that bind to its surface [1,42,45].

Several attempts have been made to find an alternative treatment: *in silico* and *in vitro* screenings to identify repurposable, existing drugs [46–48]; the combination of drugs with nanoparticles [49–53] to increase the therapeutic effectiveness; the suggestion of novel administration routes and devices [47] to increase the efficacy of drug delivery to the brain; the development of vaccines [54,55]. However, none of these treatments has reached the clinical stage yet, and the fast evolution of the disease, the dangerous nature of the amoeba, and the scarce interest of pharmaceutical companies for such a rare disease make it difficult to assess the therapeutic effect of these novel approaches *in vivo*.

In absence of a complete view of the mechanisms responsible for the pathogenicity of *N. fowleri*, in this work we present a novel therapeutic approach that focuses on targeting the amoeba’s microtubules. Microtubules are hollow tubular structures that, together with actin and intermediate filaments, constitute the cytoskeleton of a cell, and are involved in essential functions such as mechanical support, intracellular trafficking, motility, shape maintenance, and chromosome segregation during mitosis [56]. It is well known that, as part of the *Naegleria* genus, *N. fowleri* has the peculiarity of not having interphase microtubules [57]. The amoeba exhibits microtubules only in two specific occasions – mitosis in the trophozoite form, and to build flagella in the flagellate form – with actin filaments replacing them in shape maintenance, cell motility and cellular trafficking [1,58].

**Fig 2:**
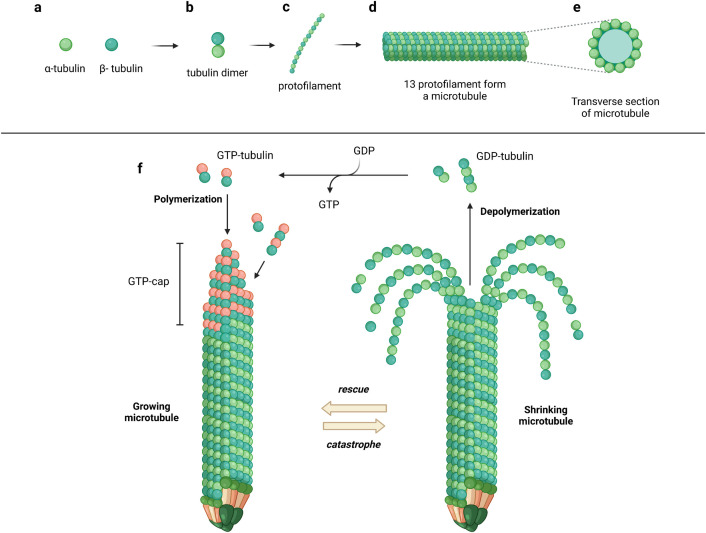
Assembly process and dynamic instability of microtubules. (a) Tubulin dimer formation involves alpha and beta tubulin subunits (dark and light green) combining to form tubulin dimers. (b) Protofilament assembly sees these dimers align head-to-tail, extending into a protofilament. (c) Microtubule construction occurs as thirteen protofilaments come together in a cylindrical formation, held stable by lateral and longitudinal interactions. (d) GTP cap and stability are achieved when the growing microtubule end is capped with GTP-bound tubulin (red), which promotes elongation. (e) Hydrolysis and catastrophe happen as GTP is converted to GDP, causing instability that leads to rapid depolymerization (or ‘catastrophe’). (f) Rescue and regrowth occur when new GTP-tubulin is added, rescuing the structure by reestablishing the cap and allowing growth to resume, vital for microtubule functions in cellular processes. Image created using Biorender.com.

Furthermore, microtubules are polymeric structures whose monomer is the tubulin dimer, that presents an α and a β subunit (Fig 2). What makes *N. fowleri*’s microtubules even more specialized is that the mitotic and the flagellate microtubules are built from two separate sets of dimers that never mix, meaning that their inhibition cannot be recovered by any other cellular actor.

Microtubules’ dynamicity and structural integrity are regulated by the GTP bound to the β subunit. However, microtubules can also be stabilized or destabilized in a controlled manner by administering small molecules, known as microtubule-targeting agents (MTAs). MTAs bind to the tubulin dimer in specific pockets [59–61], inducing conformational changes in the dimer’s structure. There are seven binding pockets for MTAs on the tubulin dimer: the Colchicine site, the Taxane site, the Vinca alkaloids site, the Peloruside site and the Maytansine site on the β monomer, the Pironetin site on the α monomer, and the and Gatorbulin-1 site at the intradimer junction between the α and the β subunit [62–64]. MTAs have been clinically used to treat several conditions since their discovery in modern medicine, more than 60 years ago [65], and their pharmacological properties are well-studied. A study by Velle et al. [57], published in 2021, suggested the possibility of employing this class of compounds as a novel treatment for PAM. Colchicine, Nocodazole, Vinblastine, Oryzalin and Docetaxel were tested *in vitro* on a close relative of *N. fowleri, Naegleria gruberi,* showing that Docetaxel was not able to bind to the amoeba’s tubulins, while the other compounds had little to no efficacy. The reason for these results was not addressed, and the lack of other studies on the use of MTAs on *N. fowleri* prompted the analysis of the amoebic tubulins’ MTA binding sites to evaluate the hypothesis of MTAs repurposing. Given the recent discovery of the Gatorbulin-1 site, and our focus on repurposing existing and clinically approved drugs, this seventh site was not taken into consideration in this study, and its analysis suggested for future studies.

Given the functions of microtubules, the amino acid sequences of the tubulin monomers are generally highly conserved across species. However, the analysis of *N. fowleri*’s sequences compared to other organisms revealed a high grade of divergence [57], which is more marked for the mitotic tubulins, sharing the 58% of sequence similarity to animal and human tubulins in contrast with the 85% of the flagellate tubulins. This divergence suggests two promising aspects. First, the amino-acid sequence of the amoeba’s tubulins is highly optimized to perform specific functions, for both the flagellate and the mitotic set. If the amoeba adapted to the treatment-induced inhibition by exhibiting mutations in its tubulin sequences, these mutations would likely result in a loss of functionality, and eventually apoptosis. Second, the differences between the amoebic sequences and those of human tubulins may show an altered interaction landscape at known binding sites, potentially allowing for the design of tubulin targeting agents specific for the amoeba and ideally harmless for the host. Taken together, these aspects *make N. fowleri*’s tubulins an ideal target to treat the infection. Given that the only form found during infection is the trophozoite, the preferred target of this therapy are the mitotic tubulins.

In this work we leveraged *in silico* approaches to actively contribute to the drug design process, with the long-term goal of developing treatments that could potentially eradicate *Naegleria fowleri* infections. Our approach was grounded in the idea that tubulin sequences are highly conserved across eukaryotes, allowing us to confidently predict the three-dimensional structures of tubulin and its drug-binding sites. At the same time, these sequences can exhibit enough variations to enable the design of drugs that target specific tubulins, such as those of *Naegleria*. A similar strategy has proven successful in the design of drugs for the βIII isotype of tubulin, which showed efficacy against a βIII over-expressing strain of breast cancer in mice. [66] That strategy can be considered as a proof-of-principle for the current study, and together with this work a template for designing drugs for other parasite diseases. The insights gained from this study could lay the groundwork for experiments needed for further validation and testing, advancing us closer to effective treatments.

In this paper, we present a comprehensive workflow to explore the potential of tubulin-targeting agents as a novel therapeutic strategy against *Naegleria fowleri* infection.

**Section 2** outlines the methodologies used to ensure robust and reliable predictions across different platforms. **Section 3** presents the results of the study, with the most relevant ones regarding the consensus docking approach, that was employed using multiple programs – MOE, AutoDock, Vina – to model the interactions between selected Colchicine analogs and their tubulin-binding sites from both *N. fowleri* and humans.

In **Section 4** we discuss the implications of these findings for drug repurposing and design, focusing on current limitations and future evolutions of the study.

Finally, in **Section 5**, we summarize our conclusions and outline the next steps for experimental validation, highlighting the significance of these computational insights in guiding future *in vitro* and *in vivo* studies. Through these sections, the paper provides a framework for the development of more targeted treatments for *N. fowleri* infections.

## 2. Materials and methods

### 2.1. Mutation analysis and comparative sequence tools for *Naegleria fowleri* tubulins

To assess the divergence between *N. fowleri* tubulins and those of other organisms, we performed a sequence comparison focusing on mutations within the six microtubule-targeting agent (MTA) binding sites. Tubulin sequences were retrieved from UniProt and supplemented by data from Velle et al. [57], distinguishing between *N. fowleri*’s flagellar and mitotic tubulins. The UniProt accession codes are provided in Supporting Information, Tables S2 and S3.

Residues constituting the tubulin binding sites were identified through a review of literature and analysis of the sequence sections of Protein Data Bank (PDB) entries of MTAs co-crystallized with tubulin. Specifically, the binding sites for Taxane, Colchicine, and Vinblastine were referenced from Huzil et al. [67], the Maytansine site from Prota et al. [68], the Peloruside/Laulimalide site from PDB entries 4O4H and 4O4J [69], and the Pironetin site from PDB entry 5LA6 [70].

For the comparative analysis, we utilized NCBI’s BLASTP [71] to measure divergence between *N. fowleri* tubulins (both flagellate and mitotic) and those of various organisms across the five kingdoms of life. Multiple sequence alignment against human and bovine tubulins was conducted using Clustal Omega [72], employing the PAM250 method to classify the conservation of the substitutions. Additional data on the effects of point substitutions were obtained from the Tubulin Mutation Database [73,74].

### 2.2. Homology modeling and validation

Homology modeling [75–78] was used to generate the 3D structures of *Naegleria fowleri* mitotic tubulins, as their experimental structures are unavailable. The process involved aligning the amoeba’s tubulin sequences with a template from the X-ray structure of colchicine-bound tubulin from *Ovis aries* (PDB: 5EYP), selected based on sequence similarity, resolution, and model quality.

Using Chemical Computing Group’s MOE, the template structure was prepared by removing non-relevant components, capping missing atoms at the termini, and using loop building to correct missing internal residues. Amber10 [79,80] was applied to address incorrect charges, and MOE’s Protonate 3D was used to optimize hydrogen positioning and calculate partial charges. The system underwent tethered minimization to relieve any structural strain.

MOE’s Homology Model application generated 25 models for each tubulin dimer, with sequences α5134 and β5966 forming one dimer and α7486 and β3784 forming the second. The models were evaluated based on geometrical and energetic parameters, followed by refinement steps, including loop remodeling and minimization. The final models were compared to the template using RMSD to verify accuracy. All steps were carried out using MOE’s default parameters unless otherwise specified.

Validation of the models was performed using tools from the UCLA-DOE’s SAVES server: ERRAT [81], Verify3D [82], WHATCHECK [83] and PROCHECK [84]. These tools ensured the reliability and quality of the models for further computational studies, such as docking simulations.

### 2.3. Selection of therapeutic agents targeting *Naegleria fowleri*

Given the necessity for therapeutic agents to efficiently cross the blood-brain barrier (BBB) to treat the infection [22,85], we prioritized compounds capable of achieving minimum inhibitory concentrations in the brain without necessitating high doses, which could be potentially toxic for patients.

**Fig 3:**
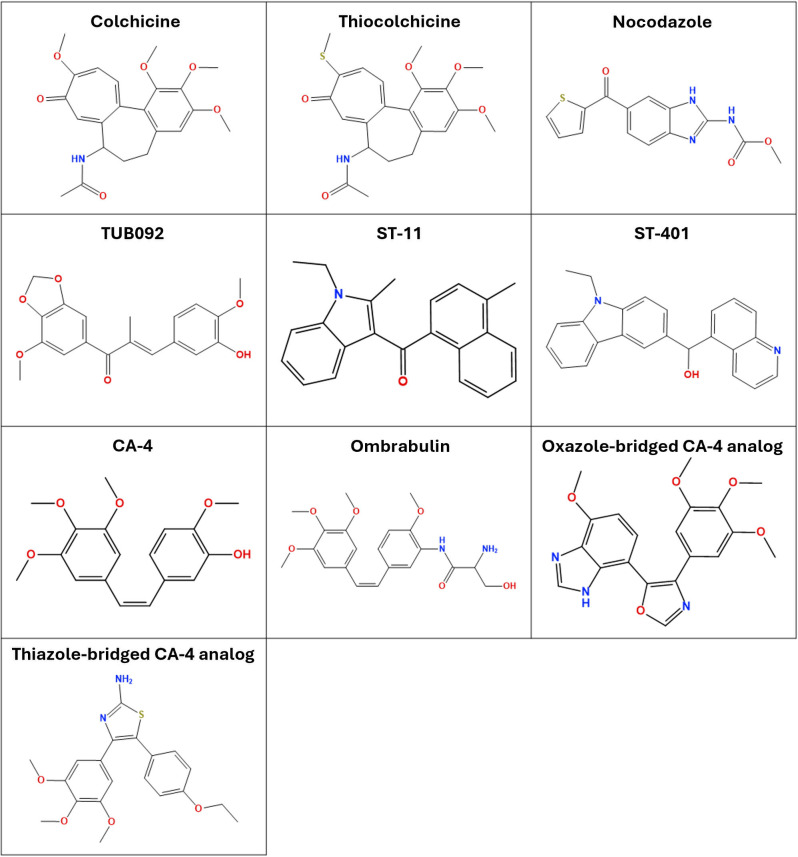
Colchicine site-targeting ligands selected for the docking simulations.

For our study, we focused on identifying microtubule-targeting agents (MTAs) that can passively permeate the BBB. We reviewed colchicine analogs developed over the past 15 years, including those approved for use or under investigation, that have demonstrated capability to cross the BBB. We identified four classes of colchicine site-targeting compounds for further analysis: ST-11, ST-401, Combretastatins and Chalconoids. The selected compounds’ chemical structures can be observed in Fig 3.

**ST-11** [86] and **ST-401** [87,88] are recently designed BBB-crossing compounds, currently in pre-clinical testing. ST-11, a 2-aroylindole derivative, is noted for its resistance to P-gp induced multidrug resistance – the most common in BBB penetration – and binds preferentially to free tubulin dimers. Interestingly, its binding is fully reversible within 24 hours of administration [86], resulting in low toxicity for the patients. ST-401 is a carbazole derivative that incorporates a quinoline moiety in its structure. Similarly to ST-11, its binding to tubulin is fully reversible, although having a milder effect compared to ST-11 and Nocodazole. [87]

**Chalconoids** [89] and **Combretastatins** [90] are two families of Colchicine analogs that differ primarily in the length of the bridge connecting the two phenyl rings in their structure. They have been developed to mimic the interactions observed between colchicine and its binding site, featuring a 3,4,5-trimethoxyphenyl ring (usually referred to as A ring) and a 3-hydroxy-4-methoxyphenyl ring (usually called B ring). These agents are known for their depolymerizing activity, and have been extensively studied for their efficacy and potential modifications [89–94].

Despite their inability to cross the BBB, Colchicine, Thiocolchicine and Nocodazole were included in our simulations as they are among the most potent ligands for the identified target. Thiocolchicine was considered also for its potential in rational derivatization [95].

The final set of ligands included: Colchicine, Thiocolchicine, Nocodazole, Combretastatin A-4 (CA-4), Ombrabulin, TUB092, ST-11, ST-401, an oxazole-bridged CA-4 analog, corresponding to compound 5 in Zhou et al. [92], and the best thiazole-bridged CA-4 analog from Ohsumi et al [93].

### 2.4. Molecular docking simulations

Molecular docking was used to model the interaction between ligands and tubulin, with receptors prepared from two *Naegleria fowleri* mitotic tubulin homology models (from PDB: 5EYP), and nine human tubulin models obtained from Vottero et al. [96] SAVES validation scores for these structures can be found in Supporting Information, Table S4.

The ligands were prepared in MOE, following structural corrections, partial charge calculations using AM1-BCC, and energy minimization. For AutoDock4 and Vina, receptors and ligands were converted to PDBQT format, with ions and ligands removed except for GTP near the colchicine site, using AutoDockTools [97]. A detailed overview of the files preparation can be found in the Supporting Information.

The search space for docking was defined uniquely for each program. In MOE, target residues were grouped into an *atom set*, while AutoDock4 and Vina used a 3D box centered on the colchicine site, defined with AMDock software [98] (details in Supporting Information). A consensus docking approach [99,100] was employed, combining results from MOE [101], AutoDock4 [97], and Vina [102,103] to mitigate the limitations of individual programs.

Benchmarking was done by docking Colchicine, Combretastatin A-4, and TUB092 to human tubulins using known native poses from PDB entries (5EYP [104], 5LYJ [69], and 5JVD [105], respectively). Poses were compared with the native pose using DockRMSD [106], with an RMSD threshold of 2 Å. For docking runs exceeding 2 Å, up to 10 attempts were made, increasing the threshold to 2.5 Å if necessary.

For the final nine Colchicine analogs, consensus was established by running each docking program at least three times per ligand-receptor pair. Consistency was checked by comparing binding energy scores (within 0.1 kcal/mol) and verifying pose similarity in the expected binding region using AutoDockTools or MOE. The best poses were selected based on their alignment in the colchicine pocket and the placement of critical functional groups, ensuring that they aligned with homologous regions in the experimental structures. Detailed description of the docking methods is included in the Supporting Information section.

### 2.5 Binding sites interaction landscape analysis method

MOE’s *Surfaces and Maps* application was used to generate both electrostatic and non-bonded interaction maps for the template structure (5EYP) and the amoeba’s Colchicine sites. These maps help visualize the three-dimensional areas within the pocket that favor hydrophobic or hydrophilic interactions, reflecting how sequence variations could affect the site’s interaction landscape.

To inspect the amoeba’s site properties and how its sequence variations could impact on the binding of Colchicine analogs, its interaction maps were compared to those for the Colchicine site of the homology modelling template and the main differences annotated. These maps however depend on the conformation of the binding site. Since the present work used rigid molecules and the colchicine site can accommodate ligands with varying shapes and sizes, biases in the computed maps were evaluated by comparing 5EYP’s maps with those of a CA-4-bound tubulin structure (5LYJ) and a chalcone-bound tubulin structure (5JVD). The results of this comparison are discussed in the Supporting Information and graphically displayed in Figs S1 and S2.

## 3. Results

### 3.1. *N. fowleri* tubulins show similar divergence from both animal and plant tubulins

The alignment of *Naegleria fowleri*’s tubulin sequences with the non-redundant BLASTP database verified significant divergence from the typical sequence conservation observed in this protein family, echoing findings by Velle et al. [57] and Herman et al. [43]. The closest sequences to *N. fowleri*’s mitotic tubulins were from *Naegleria gruberi*, exhibiting an 89% sequence identity, while animal and plants share only up to the 65%. This suggests *N. gruberi* could serve as a safer substitute for *N. fowleri* in laboratory tests, without compromising result relevance.

The homology modelling template search pinpointed the tubulins from *Sus scrofa, Bos taurus*, and *Ovis aries* as well as those from *Chlamydomonas reinhardtii* and *Toxoplasma gondii* as the most similar experimentally determined structures to *N. fowleri*. These organisms exhibited an average sequence identity of 65% and 62% to the amoeba’s mitotic β-tubulin isotypes 3784 and 5966, respectively, and 59% and 56% to α-tubulins 7486 and 5134, respectively. Complete results are detailed in Table S5 and S6 of the Supporting Information.

### 3.2. *N. fowleri* tubulin binding sites exhibit significant sequence differences from animal and human tubulins

**Fig 4:**
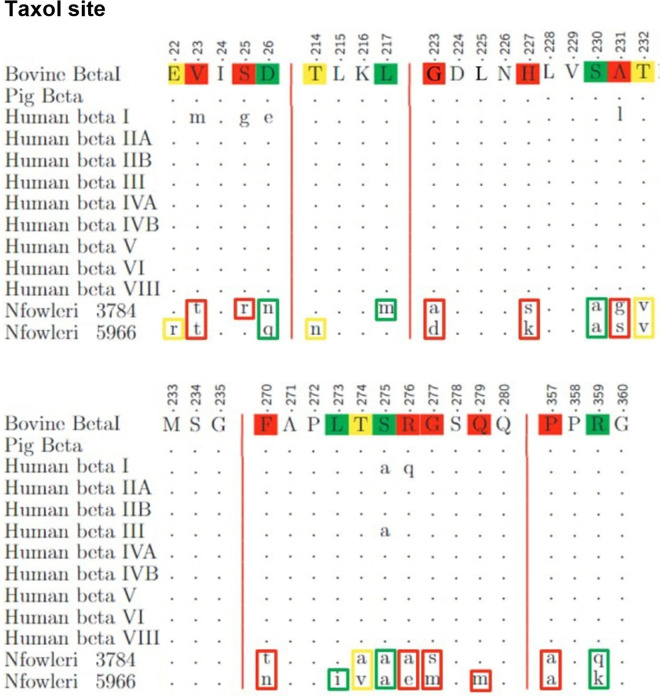
Alignment of the Taxane binding pocket sequences.

**Fig 5:**
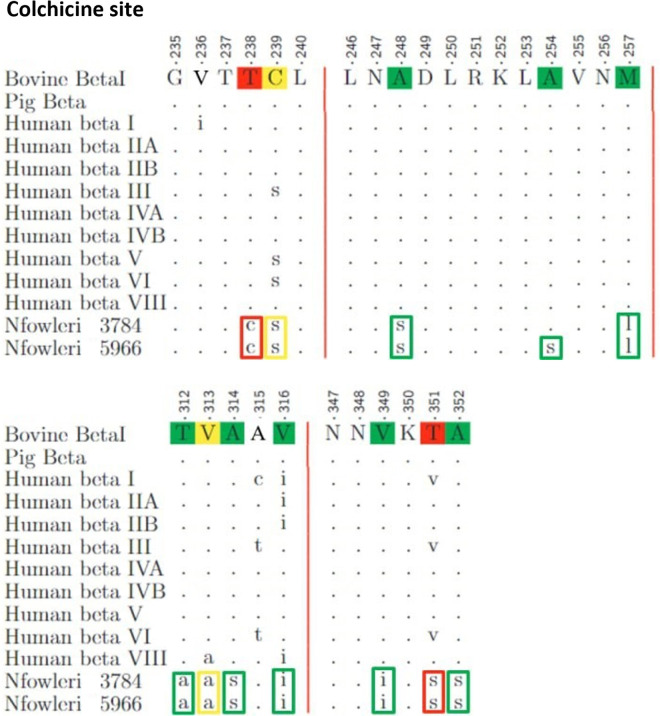
Alignment of the Colchicine binding pocket sequences.

**Fig 6:**
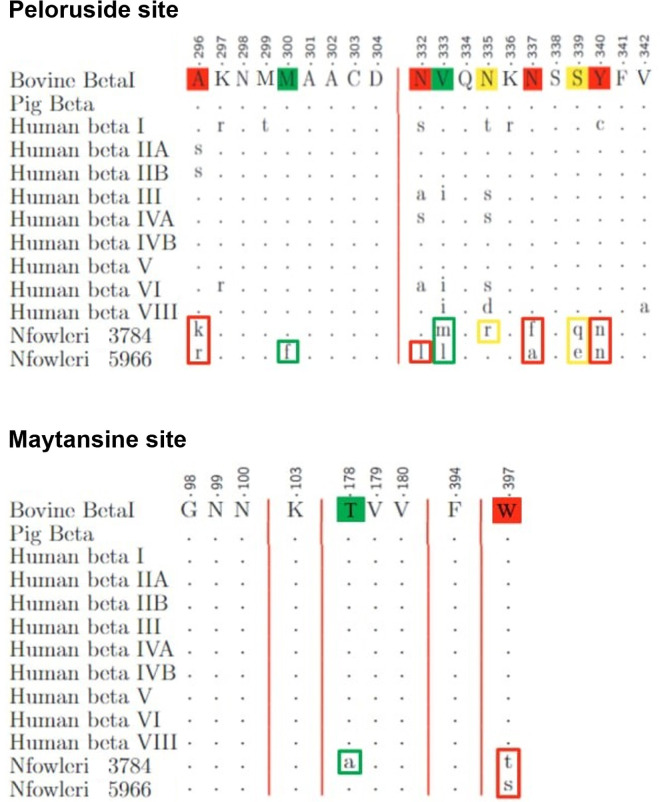
Alignments of the Peloruside and Maytansine binding pocket sequences.

**Fig 7:**
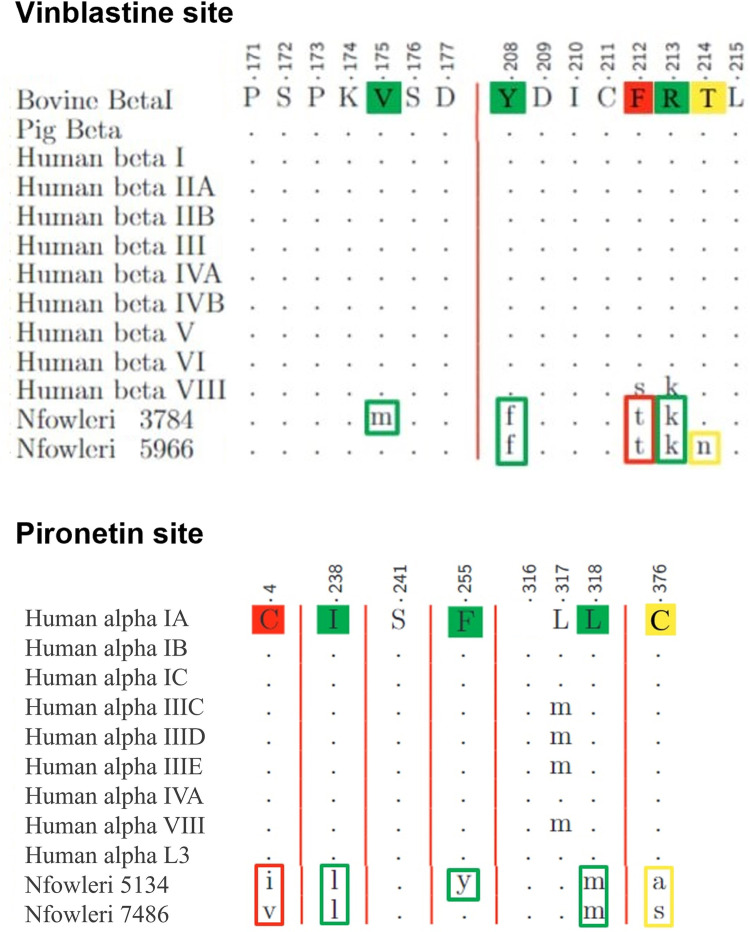
Alignments of the Vinblastine and Pironetin binding pocket sequences.

**Fig 8:**
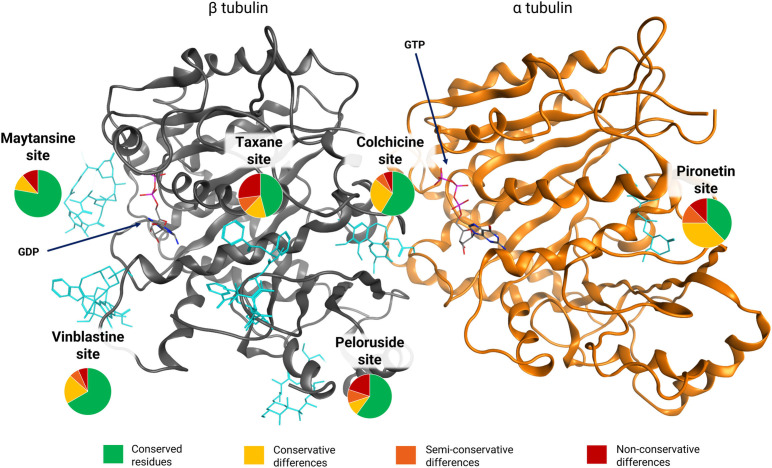
Pie charts depiction of the substitutions in each of the six tubulin binding sites of *N. fowleri,* compared to the animal sequences. The tubulin dimer displayed corresponds to *N. fowleri*’s mitotic dimer 5134-5966. Each pie chart is placed in correspondence of the related binding site, identified by the bound ligands. The pie charts are color-coded according to the legend at the bottom of the Figure.

Figs 4 to 8 illustrate the results of the alignment of microtubule-targeting agents (MTAs) binding pocket sequences. Each Fig organizes the sequences in rows labeled on the left, with the reference sequence displayed in uppercase letters at the top. Residues identical to the reference are marked with dots, while mismatches appear in lowercase letters. The degree of similarity for each modified residue is color-coded: green for conservative differences, yellow for semi-conservative differences, and red for non-conservative differences.

The analysis of mutations across the binding sites revealed varying levels of conservation in the amoeba. The Taxane site shows a composition of 6 conservative, 4 semi-conservative, and 10 non-conservative mutations, contributing to 54% of the site changes, as depicted in Fig 4. The Colchicine site, shown in Fig 5, includes 8 conservative, 2 semi-conservative, and 2 non-conservative mutations, accounting for 34.5% of the site. The Peloruside/Laulimalide site, represented in Fig 6, contains 2 conservative, 2 semi-conservative, and 4 non-conservative mutations, totaling 40% of the site. In the same Fig, the Maytansine site features 1 conservative and 1 non-conservative mutation, which corresponds to 22.2% of the site. Fig 7 highlights the Pironetin and Vinblastine sites. The former has 3 conservative, 1 semi-conservative, and 1 non-conservative mutations, making up 62.5% of the site, whereas the latter consists of 3 conservative, 1 semi-conservative, and 1 non-conservative mutations, comprising 33.3% of the site.

This detailed comparative analysis underscores the heterogeneity in sequence conservation among MTAs binding sites and highlights the potential for differential impacts on drug binding efficacy.

### 3.3. *N. fowleri* mitotic tubulins contain residues potentially linked to resistance against taxol, epothilone a, and dinitroaniline

Tables 1 and 2 showcase the results of the distal sequence alteration analysis in *Naegleria fowleri*’s mitotic tubulins. The substitutions listed are those that perfectly match entries in the Tubulin Mutations Database. Additional substitutions were identified in our analysis, although discrepancies between these and known tubulin mutations preclude reliable predictions about their effects. However, due to their potential significance in Taxane resistance, certain non-matching substitutions are reported in Table 3, which we advise be interpreted less confidently. Overall, these results suggest the presence of residues responsible for resistance against Dinitroaniline compounds, as well as against Taxane site-targeting compounds such as Taxol and Epothilone A, making these two sites unsuitable candidates for a MTA-based treatment.

**Table 1:** Alterations in *N. fowleri* mitotic α tubulins matching the tubulin mutations database entries for drug resistance or sensitization.

Known Mutation	*N. fowleri* α 5134	*N. fowleri* α 7486	Expected Effect
F49C	✓	conserved	Dinitroaniline resistance
F52L	✓	conserved	Dinitroaniline resistance
F52Y	conserved	✓	Dinitroaniline resistance
S165T	✓	✓	Dinitroaniline resistance
S178T	conserved	✓	Dinitroaniline resistance
I235V	✓	✓	Dinitroaniline resistance
V252L	conserved	✓	Dinitroaniline resistance
H283Y	✓	✓	Colcemid and Vinblastine resistance
A295V	✓	✓	Dinitroaniline resistance

**Table 2:** Alterations in *N. fowleri* mitotic β tubulins matching the tubulin mutations database entries for drug resistance or sensitization.

Known Mutation	*N. fowleri* β 3784	*N. fowleri* β 5966	Expected Effect
E198Q	conserved	✓	Benomyl and Carbendazim resistance
P220S	conserved	✓	Correlated with Colcemid resistance
N439X	✓	✓	Increased Benomyl sensitivity

**Table 3:** Non-perfect match mutations in *N. fowleri* mitotic β tubulins known to induce taxane resistance.

Known Mutation	*N. fowleri* β 3784	*N. fowleri* β 5966	Proposed Effect	Similarity to known mutation	Similarity to original residue	Similarity between non altered and known mutation
L217R	M	conserved	Taxol resistance,not implicated in binding	Complete dissimilarity	Strong similarity	Complete dissimilarity
A231T	G	S	Taxol resistance, hypersensitive to destabilizing compounds	Complete dissimilarity	Complete dissimilarity	Strong similarity
F270V	T	N	Taxol resistance	Complete dissimilarity	Complete dissimilarity	Weak similarity
T274I	A	V	Epothilone resistance, reduced sensitivity to Taxol	Complete dissimilarity	Weak similarity	Complete dissimilarity
R282N	H	S	Epothilone resistance, reduced sensitivity to Taxol	Complete dissimilarity	Complete dissimilarity	Weak similarity
R306C	T	M	Taxol resistance	Complete dissimilarity	Complete dissimilarity	Complete dissimilarity
A364T	S	S	Taxol resistance	Strong similarity	Strong similarity	Strong similarity
Y442C	E	Q	Epothilone A resistance	Complete dissimilarity	Complete dissimilarity	Complete dissimilarity

The comparative analysis of the amoeba’s tubulins against those from various organisms revealed also that mutations like *A248S, M257L, V313A, A314S,* and *V349I* are shared by *N. fowleri, N. gruberi* and *C. autumnale*, with additional mismatches such as *V361T* in *N. gruberi* and *V361M* in *C. autumnale*, and *A352S* for both amoebae compared to *A352T* in *C. autumnale*. The results of the analysis are detailed in Table 4. However, none of the identified substitutions appears in the Tubulin Mutations Database, leaving their role in Colchicine resistance speculative and warranting for further investigation.

**Table 4:** Comparative analysis of colchicine-binding regions across different organisms.

Organism	Number of residues NOT binding to colchicine	Number of residues binding within 4.5 Å of colchicine	Mutations
Human *β*I	0	0	
Human *β*IIA	0	1	V316I
Human *β*III	0	2	C239S, T351V
*N. fowleri* mitotic	2	10	C239S, A248S, M257L, T312A, V313A, A314S, V316I, V349I, T351S, A352S
*N. gruberi* flagellar	1	8	A248S, M257L, V313A, A314S, V316M, V349I, T351S, A352S
*N. gruberi* mitotic	1	10	C239S, A248S, M257L, T312A, V313A, A314S, V316T, V349I, T351S, A352S
*I. scapularis*	0	2	A315T, V316I
*P. parsiana*	0	10	V236I, A248S, M257L, V313C, A314S, A315C, V316M, V349I, T351A, A352S
*A. mellifera*	1	8	V236I, L240F, A248T, M257L, A315V, V316I, V349I, K350Q
*D. melanogaster*	0	1	V316I
*C. autumnale*	1	7	V236A, A248S, M257L, V313A, A314S, V316M,A352T

N. = *Naegleria*, I. = *Ixodes*, P. = *Phytophthora*, A. = *Apis*, D. = *Drosophila*, C. = *Colchicum.* The mutations are defined with regards to human β IV tubulin sequence. Table adapted from Luduena et al [107].

### 3.4. *Ovis aries* tubulin structure (PDB ID: 5EYP) is the optimal template for homology modeling of *N. fowleri* tubulins

Selected templates included the electron microscopy-determined microtubule structures of *Sus scrofa* (PDB IDs 3J6F and 6EVZ), the X-ray crystallography-determined structures of *Ovis aries* tubulin (PDB ID 3RYC) and its free form (PDB ID 4DRX), along with the electron microscopy-determined microtubule structures of *T. gondii* (PDB ID 7MIZ) and *C. reinhardtii* (PDB ID 7KZO). Additionally, for the Colchicine binding site, which requires a bound ligand to be formed, the free tubulin dimer of *Ovis aries* with bound colchicine (5EYP) and the tubulin structure of *Bos taurus* with bound colchicine (1SA0) were chosen. Table 5 presents a detailed comparison of these templates, including resolution, R-free values, the number of missing residues, and the year of submission to the PDB.

**Table 5:** Detailed assessment of structural quality and relevance of homology modelling templates.

PDB ID	Resolution (Å)	R-free	Missing Residues	Year
3J6F	4.9	/	11 (chain A)	2014
6EVZ	3.8	/	19 (chain A) 16 (chain B)	2017
3RYC	2.1	0.201	20 (chains A/C) 13/14 (chains B/D)	2011
4DRX	2.2	0.194	6/16 (chains A/C) 7/0 (chains B/D)	2012
7MIZ	3.4	/	25 (chain A) 23 (chain B)	2021
7KZO	3.3	/	21 (chain A) 17 (chain B)	2021
5EYP	1.9	0.208	23 (chain A) 20 (chain B)	2016
1SA0	3.6	0.249	24/29 (chains A/C) 26 (chains B/D)	2004

Models missing the R-free value are electron microscopy (Cryo-EM) determined structures, whose R-free is not indicated in the PDB database.

The template chosen for modeling the colchicine site was 5EYP, favored for its significantly finer resolution compared to 1SA0, enhancing the precision of the homology models. Among the GTP-bound structures, 4DRX and 3RYC were specifically chosen to model both free and microtubule-associated mitotic tubulins, respectively. These choices were influenced by their superior resolution and fewer missing residues compared to the other templates.

### 3.5. UCLA’s SAVES server confirms the quality and robustness of *N. fowleri* homology models

**Fig 9:**
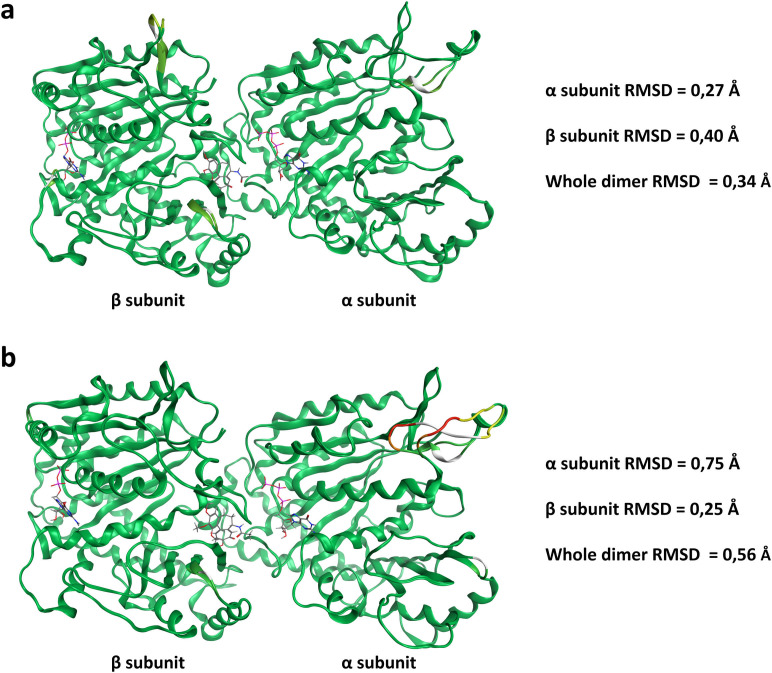
RMSD of the homology models of the two mitotic tubulin isotypes for *N. fowleri* in comparison to the template used (5EYP). (a) Superimposition of the mitotic dimer 5134−5966 with 5EYP. (b) Superimposition of the mitotic dimer 7486−3784 with 5EYP. Both images were generated using MOE. White regions of the structures identify residues on the template that are matched with a gap in the *N. fowleri* sequences. The RMSD is color coded in shades of green; the darker the color, the lower the value. One may observe regions with yellow, orange and red residues on the α subunit of dimer 7476−3784. These identify regions with local RMSD greater than 3 Å. In particular, the yellow regions have an RMSD between 3.8 and 4.5 Å, the orange region and RMSD of 5.37 Å, and the red region an RMSD of 8.4 Å. The RMSD values for each dimer and its subunits can be observed on the right of the related image. Overall, the structure of the amoeba’s mitotic tubulins is highly conserved compared to the template used.

The validation results for the models obtained through homology modelling are summarized in Table 6. Each model exhibited robust ERRAT scores, with the lowest being 86.8% for dimer 5 from template 4DRX while the others hovered around 90%, indicating that the structural errors within the models are acceptably low. The Verify3D analysis confirmed success for all models, affirming the accuracy of their spatial configurations. Procheck analysis, which evaluates *phi-psi* outliers in the structure, was successful for all the models. Indeed, when present, residues not in the most favored regions of the Ramachandran plot were found in additional favored regions, with none of the models showing residues with unfavorable *phi-psi* angles. The RMSD of the models obtained from 5EYP in comparison to their template can be observed in Fig 9.

**Table 6:** Validation scores for the homology models derived from templates 5EYP, 4DRX, and 3RYC.

Validation method	5EYP Dimer 5	5EYP Dimer 7	4DRX Dimer 5	4DRX Dimer 7	3RYC Dimer 5	3RYC Dimer 7
ERRAT	90.4%	93.3%	86.8%	88.9%	88.6%	90.8%
Verify 3D	92.2%	94.3%	89.0%	95.9%	92.4%	94.0%
Ramachandran	89.4%	90.1%	85.9%	85.2%	84.1%	86.0%

### 3.6. Docking programs agree that colchicine is the best binder for human tubulins

The results of the docking for the chosen compounds on the human tubulins are detailed in Tables 7–9. These tables display binding energies calculated in kcal/mol, highlighting the best (lowest energy) pose for each ligand-receptor complex.

**Table 7:** MOE’s binding energy scores for colchicine site-targeting compounds on the nine human tubulins models. Values given in kcal/mol.

Ligand	βI	βIIa	βIIb	βIII	βIVa	βIVb	βV	βVI	βVIII
Colchicine	−10.4	−10.5	−10.4	−10.3	−10.6	−10.8	−10.4	−10.6	−10.5
Thiocolchicine	−9.9	−10.0	−10.2	−10.1	−10.3	−10.5	−10.1	−10.3	−10.3
Nocodazole	−7.6	−7.4	−7.4	−7.3	−7.4	−7.7	−7.2	−7.2	−7.3
TUB092	−8.8	−8.8	−8.8	−8.4	−7.8	−8.4	−7.6	−8.2	−8.3
ST-11	−7.7	−7.6	−7.8	−7.6	−7.7	−7.7	−7.7	−7.6	−7.8
ST-401	−8.0	−7.7	−8.1	−8.0	−7.9	−7.8	−7.8	−8.1	−7.7
CA-4	−8.4	−7.7	−8.4	−8.4	−7.7	−7.8	−7.3	−8.7	−8.6
Ombrabulin	−9.0	−8.9	−9.3	−8.9	−8.9	−9.0	−8.7	−8.9	−9.0
Oxazole-bridged CA-4	−8.7	−8.6	−8.5	−8.7	−8.5	−8.6	−7.9	−9.2	−8.7
Thiazole-bridged CA-4	−9.1	−9.4	−9.3	−9.2	−8.7	−9.4	−9.3	−9.3	−9.1

**Table 8:** Vina’s binding energy scores for colchicine site-targeting compounds on the nine human tubulins models. Values given in kcal/mol.

Ligand	βI	βIIa	βIIb	βIII	βIVa	βIVb	βV	βVI	βVIII
Colchicine	−11.0	−10.9	−10.9	−11.4	−11.1	−11.0	−11.7	−11.5	−11.0
Thiocolchicine	−10.5	−10.7	−10.2	−11.0	−10.7	−10.7	−10.9	−10.7	−10.5
Nocodazole	−8.3	−8.2	−8.2	−8.3	−8.4	−8.3	−8.2	−8.1	−8.3
TUB092	−9.4	−9.5	−9.5	−9.5	−9.4	−9.4	−9.5	−9.6	−9.4
ST-11	−9.9	−10.6	−10.2	−10.4	−10.7	−10.1	−10.7	−9.4	−10.4
ST-401	−10.2	−10.3	−10.3	−9.8	−10.1	−10.1	−9.6	−9.9	−10.3
CA-4	−7.3	−7.4	−7.4	−7.5	−7.4	−7.4	−7.5	−7.4	−7.5
Ombrabulin	−8.4	−8.4	−8.4	−8.5	−7.8	−8.1	−8.5	−8.5	−8.4
Oxazole-bridged CA-4	−8.9	−8.8	−8.7	−8.7	−8.8	−8.9	−8.7	−8.1	−8.4
Thiazole-bridged CA-4	−8.1	−8.2	−8.1	−7.8	−8.1	−8.1	−8.2	−7.8	−7.7

**Table 9:** AutoDock4’s binding energy scores for colchicine site-targeting compounds on the nine human tubulins models. Values given in kcal/mol.

Ligand	βI	βIIa	βIIb	βIII	βIVa	βIVb	βV	βVI	βVIII
Colchicine	−11.5	−11.5	−11.5	−11.6	−11.8	−11.8	−11.5	−11.8	−11.7
Thiocolchicine	−10.1	−10.0	−10.0	−10.0	−10.0	−9.8	−9.2	−10.0	−10.1
Nocodazole	−8.9	−9.0	−9.0	−8.9	−9.9	−9.1	−9.1	−9.1	−8.8
TUB092	−10.3	−10.1	−10.1	−10.3	−10.3	−10.3	−10.2	−10.1	−10.3
ST-11	−9.1	−8.9	−8.9	−9.1	−9.1	−8.9	−9.1	−9.1	−8.9
ST-401	−10.6	−10.6	−10.6	−10.5	−10.7	−10.6	−10.6	−10.7	−10.6
CA-4	−7.9	−7.5	−7.4	−8.0	−7.8	−7.6	−7.3	−8.2	−8.1
Ombrabulin	−9.4	−9.5	−9.0	−9.0	−9.0	−9.1	−9.0	−9.3	−9.4
Oxazole-bridged CA-4	−7.2	−7.0	−7.2	−7.0	−6.9	−6.9	−6.8	−7.2	−7.5
Thiazole-bridged CA-4	−7.9	−8.0	−8.0	−8.3	−7.9	−7.9	−7.7	−8.5	−8.0

Results regarding Colchicine, Combretastatin A-4 (CA-4) and TUB092 correspond to the results of the programs benchmarking process. All three docking programs successfully predicted the native poses for these compounds within a 2 Å RMSD, demonstrating their reliability in the study.

Notably, both Vina and AutoDock4 showed slightly higher binding energy values compared to MOE. Colchicine consistently emerged as the best binder in all three software programs, reinforcing its potential as a highly effective agent.

### 3.7. Colchicine analogs show comparable binding affinities for both *N. fowleri* and human tubulins

The results regarding the docking of the chosen compounds on *N. fowleri*’s mitotic tubulins are documented in Tables 10,11,12. These tables display binding energies calculated in kcal/mol, highlighting the best (lowest energy) pose for each ligand-receptor complex. Interestingly, all the compounds show binding energies comparable with those observed when docking to the human tubulins, supporting the hypothesis that the effect of the different amino acid composition is overall conservative, and suggesting that colchicine analogs should retain their ability to bind to the amoeba’s site.

**Table 10:** MOE docking results for colchicine site-targeting ligands on *N. fowleri*’s mitotic tubulins.

Compound	Avg. Human	Dimer 5	Dimer 7
Colchicine	−10,5	−11.0	−10.4
Thiocolchicine	−10.2	−11.3	−10.5
Nocodazole	−7.4	−8.3	−7.3
TUB092	−8.3	−9.0	−8.4
ST-11	−7.7	−7.8	−7.6
ST-401	−7.9	−6.3	−7.6
CA-4	−8.1	−7.9	−8.3
Ombrabulin	−9.0	−7.4	−9.5
Oxazole-bridged CA-4	−8.6	−8.8	−8.7
Thiazole-bridged CA-4	−9.2	−8.3	−8.8

**Table 11:** AutoDock Vina docking results for colchicine site-targeting ligands on *N. fowleri*’s mitotic tubulins.

Compound	Avg. Human	Dimer 5	Dimer 7
Colchicine	−11.2	−10.4	−11.5
Thiocolchicine	−10.7	−10.1	−10.2
Nocodazole	−10.4	−8.6	−8.0
TUB092	−9.5	−7.8	−9.0
ST-11	−10.3	−8.4	−8.9
ST-401	−10.1	−7.2	−8.0
CA-4	−7.4	−7.0	−7.6
Ombrabulin	−8.3	−7.7	−7.2
Oxazole-bridged CA-4	−8.7	−6.3	−8.5
Thiazole-bridged CA-4	−8.0	−6.7	−7.0

**Table 12:** AutoDock4 docking results for colchicine site-targeting ligands on *N. fowleri*’s mitotic tubulins.

Compound	Avg. Human	Dimer 5	Dimer 7
Colchicine	−11.6	−11.1	−12.1
Thiocolchicine	−10.0	−7.8	−8.6
Nocodazole	−9.1	−8.3	−8.4
TUB092	−10.1	−9.5	−9.3
ST-11	−9.0	−9.9	−9.9
ST-401	−10.6	−8.6	−8.8
CA-4	−7.7	−7.2	−7.6
Ombrabulin	−8.7	−9.0	−8.2
Oxazole-bridged CA-4	−7.1	−6.1	−8.4
Thiazole-bridged CA-4	−8.0	−6.5	−7.3

The binding energies are reported in kcal/mol. “Avg. Human” denotes the average binding energy of these compounds as determined by docking them onto nine different human tubulin models using MOE, with these averages sourced from Table 7. “Dimer 5” corresponds to the mitotic dimer 5134−5966, and “Dimer 7” to the mitotic dimer 7486−3784.

The binding energies are reported in kcal/mol. “Avg. Human” denotes the average binding energy of these compounds as determined by docking them onto nine different human tubulin models using AutoDock Vina, with these averages sourced from Table 8. “Dimer 5” corresponds to the mitotic dimer 5134−5966, and “Dimer 7” to the mitotic dimer 7486−3784.

The binding energies are reported in kcal/mol. “Avg. Human” denotes the average binding energy of these compounds as determined by docking them onto nine different human tubulin models using AutoDock4, with these averages sourced from Table 9. “Dimer 5” corresponds to the mitotic dimer 5134−5966, and “Dimer 7” to the mitotic dimer 7486−3784.

### 3.8 *N. fowleri* interaction maps reveal potential alterations in binding dynamics, while core features of the site remain conserved

The maps created for the two *N. fowleri* mitotic dimers showed nearly identical results, which was anticipated given that the Colchicine site is primarily located on the β subunit, and these sequences differ by only one residue.

**Fig 10:**
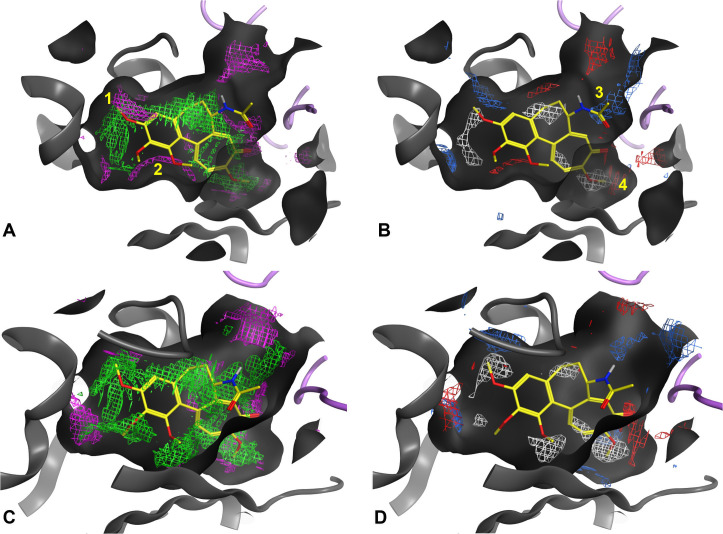
Comparison of the electrostatic and non-bonded interaction maps at the colchicine site between *N. fowleri*’s mitotic dimer 5134-5966 and PDB entry 5EYP. The key changes influencing ligand interaction are highlighted with numbers 1 to 4. SubFigs A and C represent the non-bonded interaction maps, where green areas represent the most suitable locations for hydrophobic groups, while purple areas are those for hydrophilic groups. SubFigs C and D represent the electrostatic interaction maps, where white areas represent the most suitable locations for hydrophobic groups, blue areas those for H-bond donors and red areas those for H-bond acceptors. Although referring to similar properties, it is possible to observe slight differences between the two interactions profiles because of the way each is calculated. Overall, it is reasonable to say that they provide complementary rather than redundant information.

Their comparison with 5EYP’s maps revealed that, while the core characteristics of the binding site remain predominantly hydrophobic, modifications in hydrophilic interactions occur around key areas, indicated in Fig 10 with numbers 1–4. In particular, areas 1 and 2 overlap with the methoxy groups of the TMP ring of Colchicine analogs, while area 3 and 4 do not alter significantly the landscape in their respective regions. While these modifications could potentially influence ligand binding dynamics, they should not drastically alter the binding mode observed in human tubulin, which supports the hypothesis of observing similar poses and binding affinities for the two organisms.

## 4. Discussion

### 4.1. Implications of sequence variations in *N. fowleri*’s tubulin binding sites

To assess the viability of an MTA-based therapy, we first conducted a detailed analysis of the amoeba’s tubulin sequences. Our findings highlighted the distinctiveness of *N. fowleri*, indicating that its mitotic tubulins significantly diverge from those of any other organism outside its genus. Although the mitotic sequences of *N. fowleri* appear to be distantly related to both animal and plant tubulins, their closer resemblance to plant tubulins and to those of *T. gondii* and *P. falciparum* suggests a potential for repurposing dinitroaniline agents, which have no effect on humans. [108] However, the amoeba’s tubulin binding sites present altered sequences compared to the humans, varying significantly in both nature and extent depending on the site. Furthermore, the analysis of substitutions outside of the binding sites revealed that certain residues in the amoeba could contribute to resistance against dinitroaniline [109] and taxanes, indicating these two classes of compounds as potentially ineffective. These findings are mirrored by the *in vitro* analysis of Velle et al. on *N. gruberi*, showing the inefficacy of a dinitroaniline agent (Oryzalin) and Docetaxel. The Peloruside/Laulimalide site shows substantial modifications that could compromise the effectiveness of the treatment too. On the contrary, the Maytansine site remains highly conserved, which would make treatments not able to preferentially target the pathogen’s receptor. The Vinblastine and the Colchicine sites fall in between, presenting enough conserved residues to support the repurposing hypothesis, and enough substitutions to support the selectivity requirement. The Colchicine site was chosen because of the better understanding of the mode of action of its analogues, and the greater efforts in their derivatization for CNS pathologies [86,87,90–92,110–115]. Fig 8 provides a graphical summary of the results on binding sites’ composition.

An aspect that should not be however ignored is that sequence variations may affect the structural rigidity of the amoeba’s tubulins and alter the positioning of crucial residues involved in ligand binding, thus potentially influencing the structural dynamics and contributing to drug resistance. The absence of such analysis is a limitation of the current study. We suggest it be included in future research on the matter and supported by *in vitro* experiments, for a comprehensive understanding of the impact of sequence variations on ligand binding. In the present work, a preliminary evaluation of this aspect was done by analyzing the interaction landscape of the Colchicine binding site; however, not accounting for the dynamicity of the system makes it necessary to rely on more sophisticated *in silico* techniques or lab experiments for future studies.

### 4.2. Implications of docking simulations for targeted therapy against *Naegleria fowleri*

The docking simulations conducted with the selected programs revealed that the tested Colchicine analogs exhibit similar affinities for both the amoeba’s and the human tubulins. In the docking results at the amoeba’s site, Colchicine and Thiocolchicine were the most effective binders for both MOE and Vina, while AutoDock4 scored Thiocolchicine with a worse affinity than Nocodazole, TUB092, ST-11 and ST-401.

Despite these differences, the binding scores for the amoeba’s tubulins across all three programs were similar to those obtained for the human tubulins. This observation, along with the analysis of the interaction maps at the amoeba’s binding site (Fig 10), suggests that using colchicine site-targeting compounds could be a viable therapy for PAM. Indeed, the compounds bound to the amoeba’s tubulins with a strength similar to that seen when binding to the human receptor, an aspect consistent with the results from tests on *N. gruberi.*[57]

The limit however of docking simulations is the lack of evidence on the maintenance of therapeutic efficacy – or activity – of the compounds. Indeed, while no direct evidence of Colchicine resistance has been found in *N. fowleri*, there are minimal alterations in the interaction landscape of the binding site that could potentially affect the efficacy of these compounds and could not be evaluated thoroughly because of conformation dependency. Velle et al. [57] have shown that Colchicine and Nocodazole have minimal or no impact on *N. gruberi’s* mitotic tubulins, which suggests the idea that some resistance mechanisms could have been overlooked. Consequently, Colchicine resistance mechanisms in *N. fowleri* could not be ruled out completely in our study, and further investigation should be carried out through lab experiments.

The similar binding energies of the ligands to both the amoeba’s and human tubulins highlight a crucial point for treatment efficacy: the compounds lack selectivity. This was somewhat anticipated since these compounds are effective binders for human tubulins. As several studies have shown how Colchicine derivatives can exhibit either neurotoxic or neuroprotective effects, it is necessary to consider this behaviour when designing a treatment for PAM. It is well known that Colchicine possesses a significant neurotoxic profile, primarily due to the increase of intracellular free zinc, the consequent interference with zinc axonal transport, and the production of reactive oxygen species as a result of its interaction with tubulin. [116–119] Furthermore, this ligand is involved in chemotherapy-induced cognitive impairment (CICI) [120], with its neurotoxicity selective for the hippocampal dentate granule cells. [116] Nocodazole exhibits a similar behaviour, negatively affecting axonal transport [121]. Clinical studies on CA-4 showed a transient dose-related neurotoxicity for its prodrug, CA-4 phosphate, when administered above 60 mg/m^2^ [122,123], thus suggesting a relatively safe profile below this threshold. Chalcones have been instead shown to possess neuroprotective and antioxidant properties [124,125], while there are no experimental evaluations on the neurotoxicity of ST-11 and ST-401 to this date. While these aspects point out Chalcones and Combretastatins as the most suitable options for a treatment, addressing the knowledge gap in ST-11 and ST-401 mode of action and neurotoxicity could provide novel opportunities for intervention. Nonetheless, it has been shown that brain microtubules are more stable than those in other tissues [126], a characteristic that varies with the developmental stage of the neurons and is negatively affected by increased levels of tubulin βIII isotype. [126] Additionally, colchicine site-targeting ligands show a preference for free tubulin dimers. These factors together suggest that therapies using Colchicine analogs might still be useful against the amoeba’s infection, potentially minimizing risks to the host. Early detection of the infection would however still be crucial for a positive outcome.

The modifications observed in the mitotic sequences could also be leveraged, in future studies, to develop enhanced Colchicine derivatives, able to target the amoeba’s tubulins more effectively while minimizing any undesirable effects on the host. Chalconoids and Combretastatins appear as the most promising scaffolds. Numerous analogs of these compounds have been created and assessed, and their physico-chemical characteristics facilitate their redesign to traverse the BBB. Scarce BBB permeation is one of the major limits for MTAs, and it is a crucial factor for treating this infection effectively. Poor BBB permeation is primarily due to the presence of efflux pumps (particularly P-glycoprotein) on the vessel walls of the brain capillaries, that recognize most tubulin-targeting compounds as substrates and pump them out of the brain into the bloodstream, bringing their concentration in the brain below the therapeutic threshold. Only ST-11 and ST-401 have demonstrated significant BBB permeability without modifications [86,87], which considerably narrows the options for potential candidates if relying solely on passive membrane permeation. Intranasal administration, as suggested by Baig et al. [47,48], might provide means to treat PAM without requiring BBB penetration. We speculate that this method could be applied to anti-tubulin drugs similarly to insulin [127], to support effective delivery to the brain potentially expanding the range of usable compounds.

## 5. Conclusion

*Naegleria fowleri* is undoubtedly a remarkable organism. Since its discovery in 1965, its ability to adapt and survive in extreme conditions without losing viability or pathogenicity has been extensively studied. [1] Despite numerous efforts, the precise mechanisms involved in its pathogenic behavior towards humans and animals remain elusive, though several hypotheses have been proposed. [1,5,22,41–43,45,54,58,128–130] This uncertainty can be attributed to two main factors: the expression of many potential pathogenic factors for *N. fowleri* by its harmless relatives*, Naegleria gruberi* and *Naegleria lovaniensis*, and the redundancy of cellular actors performing the same functions in this pathogen. These aspects emphasize the challenge of identifying a suitable therapeutic target, whose functions are indispensable for the amoeba yet cannot be compensated for by other cellular mechanisms.

In this work we propose the most promising target be the amoeba’s mitotic tubulins. Indeed, unlike most organisms, *Naegleria* amoebae lack interphase microtubules [57], and only synthesize them under specific conditions: to construct tail-like flagella, and during mitosis. Additionally, in *N. fowleri* flagellar and mitotic microtubules are composed of distinct sets of tubulin dimers that never mix with one another. Targeting the mitotic tubulins could therefore force the amoeba into apoptosis, as it lacks alternative mechanisms to compensate for the disruption of their function.

Microtubules are ubiquitous in eukaryotic organisms, and their polymeric structure as well as their roles in intracellular transport and mitosis are highly conserved [131]. It has long been known that the amino acid sequences of both α- and β-tubulin have been highly conserved in evolution too [132], which raises the question of the necessity for this conservation. Any mutation that might impair the α-β dimer’s ability to assemble and polymerize would likely disrupt essential microtubule functions. Additionally, both α- and β-tubulin need to bind to GTP, and importantly, the GTP attached to β-tubulin must undergo hydrolysis, contributing to the overall dynamic nature of the microtubule structure. This necessitates that only minimal changes in the tubulin amino acid sequence are tolerable to maintain functional integrity.

Over the approximately two billion years since the ancestors of *Naegleria* diverged from those of multicellular eukaryotes [133], a select number of mutations have however been accepted in tubulin, some of which affecting the binding sites for microtubule-targeting agents as observed in this organism. This has three main implications. First, drugs designed to target MTA-binding sites in *N. fowleri* are less likely to bind effectively to the corresponding sites in human tubulin, potentially reducing unwanted side effects. Second, the strong evolutionary constraints on tubulin suggest that it would be challenging for the targeted pathogen to mutate these sites and develop resistance to the drugs. Lastly, studying how a drug interacts with both the pathogen’s and human tubulin can enhance our understanding of tubulin’s structure-function relationships, which is vital for the rational design of more effective and selective therapeutic agents. The strategy presented in this manuscript could therefore also be applicable to designing treatments for other diseases such as malaria.

To assess the viability of targeting tubulin in *Naegleria fowleri*, we first conducted a detailed analysis of the amoeba’s tubulin sequences, examining both their local and broader structural properties. Our analysis revealed that *N. fowleri*’s sites present varying levels of conservation compared to the human sites, with distal residues likely to be involved in drug resistance. The full sequence analysis prompted also the observation that variations in *N. fowleri*’s sequence, especially in terms of deletions and insertions, could alter the structural rigidity of the receptor, allowing ligands to bind but significantly reducing their efficacy (as observed *in vitro* on *N. gruberi.*[57]).

Deeming the Colchicine site as the most suitable target, we selected 10 Colchicine analogs (depicted in Fig 3) for *in silico* testing, using a consensus docking protocol involving MOE, AutoDock4 and Vina. The simulations were performed on two mitotic dimers for the amoeba, and on nine human tubulin models corresponding to the combination of each human β-tubulin isotype with the human αI-tubulin isotype. The results showed similar binding energies and poses for the tested compounds on both organisms. While this suggests that colchicine analogs should retain their ability to bind to the amoeba’s site, which is supported by Velle et al. [57], on the other hand it is an indicator of poor selectivity. Although this was expected, the repurposing hypothesis of such compounds should not be disregarded: colchicine site-targeting compounds bind preferentially to free dimers, and neuronal microtubules are less susceptible to the dynamic instability process [126]. Taken together, these aspects may indicate that, although requiring perfectioning, an MTA-based treatment could still be applied with limited risks for patients.

The need to cross the blood-brain barrier (BBB) however poses a significant challenge in drug delivery, since only two of the tested compounds (ST-11 and ST-401) show good BBB permeation. While this reduces significantly the pool of compounds to select from, the delivery of therapeutics via intranasal administration has recently been proposed as a way to bypass the BBB (Baig et al. [47,48]), which was already shown to be effective for other pathologies [134].

Given the presence of sequence variations, we deem valuable to consider the rational derivatization of existing compounds to improve both their physicochemical properties and their selectivity for the amoeba. In this context, the most promising templates are combretastatins and chalconoids, whose modification can be made more effective by combining classical medicinal chemistry knowledge with our analysis method for binding sites’ interaction landscapes. Finally, since *N. fowleri* mitotic tubulins share several altered residues with the tubulin sequences of other pathogens such as *Phytophthora parsiana* and *Ixodes scapularis*, an effective MTA analog for the amoeba might also be repurposed for treating a broader range of infections. This strategy aligns with previous studies that have focused on designing versatile, repurposable compounds for a variety of pathogens and infections [107,135], making this study a corner stone in the development of promising therapies for this rare disease.

## Supporting information

S1 Table Mode of action for the drugs currently used to treat PAM. (DOCX)

S2 Table UniProt accession codes of the α tubulins sequences used. (DOCX)

S3 Table UniProt accession codes of the β tubulins sequences used. (DOCX)

S4 Table Validation scores for the human tubulins homology models. (DOCX)

S5 Table *N. fowleri*’s α tubulins alignment to *T. gondii*, *P. falciparum* and human α tubulins. (DOCX)

S6 Table *N. fowleri*’s β tubulins alignment to *T. gondii*, *P. falciparum* and human β tubulins. (DOCX)

S7 Additional information on the methods. (DOCX)

S1 Fig Comparison of the conformation of PDB entries 5EYP, 5LYJ and 5JVD. (TIF)

S2 Fig Electrostatic and non-bonded interaction maps in the colchicine site of PDB models 5EYP, 5LYJ and 5JVD. (TIF)
