# Correction: Computational discovery of potential therapeutic agents against brain-eating amoeba (*Naegleria fowleri*)

**DOI:** 10.1371/journal.pone.0343932

**Published:** 2026-02-25

**Authors:** The PLOS One Staff

There are errors in the Author Contributions. The correct contributions are:

**Conceptualization:** Jacopo Zattoni, Richard F. Ludueña, Jack A. Tuszynski

**Data curation:** Jacopo Zattoni

**Formal analysis:** Jacopo Zattoni

**Investigation:** Jacopo Zattoni, Richard F. Ludueña

**Methodology:** Jacopo Zattoni, Jack A. Tuszynski

**Project administration:** Jack A. Tuszynski

**Resources:** Jack A. Tuszynski

**Software:** Jacopo Zattoni

**Supervision:** Richard F. Ludueña, Jack A. Tuszynski

**Validation:** Jacopo Zattoni, Richard F. Ludueña

**Visualization:** Jacopo Zattoni

**Writing – original draft:** Jacopo Zattoni

**Writing – review & editing:** Jacopo Zattoni, Richard F. Ludueña, Maral Aminpour, Jack A. Tuszynski

The publisher apologizes for the error.
