# Supplementary material for: Computational discovery of potential therapeutic agents against brain-eating amoeba (Naegleria fowleri)
Source: PLoS One. 2025 Jul 11;20(7):e0327621. doi: 10.1371/journal.pone.0327621 (PMC12250431; doi:10.1371/journal.pone.0327621)
Supplement: S1 Table — (DOCX) [file pone.0327621.s001.docx]

**Table S1. Mode of action for the drugs currently used to treat PAM.**

| **Name** | **Description** | **Mode of Action** |
| --- | --- | --- |
| Miltefosine | Antileishmania, antimicrobial,  anti cancer, anti-FLA | Mitochondrial inhibition of cytochrome-c  oxidase; inhibition of phosphatidylcholine biosynthesis and of protein kinase B |
| Dexamethasone | Anti-inflammatory glucocorticoid | Decreased vasodilation and permeability  of capillaries, decreased leukocyte migration to inflammation sites |
| Azithromicin | Antibiotic for the treatment of enteric,  respiratory and genitourinary infections | Inhibition of bacterial protein synthesis and  translation |
| Rifampin | Antibiotic for mycobacterial  infections | Inhibition of bacterial DNA-dependent  RNA polymerase |
| Fluconazole | Antifungal | Steroidal inhibition in fungal cells,  inhibition of cell wall synthesis |
| Amphotericin B | Antifungal (Fungistatic or fungicidal  depending on concentration) | Binds to sterols in the membrane  of fungi disrupting the membrane |
