# Supplementary material for: Computational discovery of potential therapeutic agents against brain-eating amoeba (Naegleria fowleri)
Source: PLoS One. 2025 Jul 11;20(7):e0327621. doi: 10.1371/journal.pone.0327621 (PMC12250431; doi:10.1371/journal.pone.0327621)
Supplement: S2 Table — (DOCX) [file pone.0327621.s002.docx]

**Table S2. UniProt accession codes of the α tubulins sequences used.**

| **Organism** | **Uniprot Accession Code** |
| --- | --- |
| *N. fowleri* Flagellate α | A0A6A5C3W6 |
| *N. fowleri* mitotic α 5134 | A0A6A5BIF6 |
| *N. fowleri* mitotic α 7486 | A0A6A5BXT2 |
| *T. gondii* | B9PJD4 |
| *P. falciparum* | P14642 |
| Human α Ia | Q71U36 |
| Human α Ib | P68363 |
| Human α Ic | Q9BQE3 |
| Human α IIIc | P0DPH7 |
| Human α IIId | P0DPH8 |
| Human α IIIe | Q6PEY2 |
| Human α IVa | P68366 |
| Human α IVb | P68371 |
| Human α VIII | Q9NY65 |
| Human α L3 | A6NHL2 |

N. = *Naegleria*, T. = *Toxoplasma*, P. = *Plasmodium*
