# Supplementary material for: Computational discovery of potential therapeutic agents against brain-eating amoeba (Naegleria fowleri)
Source: PLoS One. 2025 Jul 11;20(7):e0327621. doi: 10.1371/journal.pone.0327621 (PMC12250431; doi:10.1371/journal.pone.0327621)
Supplement: S3 Table — (DOCX) [file pone.0327621.s003.docx]

**Table S3. UniProt accession codes of the β tubulins sequences used.**

| **Organism** | **Uniprot Accession Code** |
| --- | --- |
| *N. fowleri* flagellate β | A0A6A5BK53 |
| *N. fowleri* mitotic β 3784 | A0A6A5C913 |
| *N. fowleri* mitotic β 5966 | A0A6A5BVM4 |
| *T. gondii* | B9PWK0 |
| *P. falciparum* | Q7KQL5 |
| Human β I | Q9H4B7 |
| Human β IIa | Q13885 |
| Human β IIb | Q9BVA1 |
| Human β III | Q13509 |
| Human β IVa | P04350 |
| Human β IVb | P68371 |
| Human β V | P07437 |
| Human β VI | Q9BUF5 |
| Human β VIII | Q3ZCM7 |

N. = *Naegleria*, T. = *Toxoplasma*, P. = *Plasmodium*
