# Supplementary material for: Computational discovery of potential therapeutic agents against brain-eating amoeba (Naegleria fowleri)
Source: PLoS One. 2025 Jul 11;20(7):e0327621. doi: 10.1371/journal.pone.0327621 (PMC12250431; doi:10.1371/journal.pone.0327621)
Supplement: S4 Table — (DOCX) [file pone.0327621.s004.docx]

**Table S4. Scores obtained from the validation process of the human tubulins homology models, using UCLA’s Saves and QMEAN.** According to these tools’ guidelines, all models are of excellent quality. Table taken from Vottero et al. [1].

| **Validation method** | **human**  ***β*I** | **human**  ***β*IIa** | **human**  ***β*IIb** | **human**  ***β*III** | **human**  ***β*IVa** | **human**  ***β*IVb** | **human**  ***β*V** | **human**  ***β*VI** | **human**  ***β*VIII** |
| --- | --- | --- | --- | --- | --- | --- | --- | --- | --- |
| ERRAT | 94*.*38 | 94*.*01 | 93*.*65 | 94*.*26 | 94*.*25 | 94*.*26 | 94*.*37 | 94*.*21 | 94*.*26 |
| Verify3D | 99*.*42% | 99*.*31% | 99*.*42% | 99*.*19% | 99*.*42% | 99*.*54% | 98*.*27% | 99*.*65% | 99*.*54% |
| Ramachandran | 92*.*2% | 91*.*1% | 91*.*1% | 91*.*4% | 91*.*8% | 91*.*7% | 91*.*0% | 91*.*1% | 91*.*5% |
| QMEAN4 | 0*.*73 | 0*.*73 | 0*.*73 | 0*.*73 | 0*.*73 | 0*.*73 | 0*.*73 | 0*.*72 | 0*.*73 |
| QMEAN6 | 0*.*71 | 0*.*71 | 0*.*71 | 0*.*70 | 0*.*70 | 0*.*71 | 0*.*71 | 0*.*71 | 0*.*71 |

**Reference**

1. Vottero P, Wang Q, Michalak M, Aminpour M, Tuszynski JA. Computational Analysis and Experimental Testing of the Molecular Mode of Action of Gatastatin and Its Derivatives. Cancers. 2023;15: 1714. doi:10.3390/cancers15061714
