# Supplementary material for: Computational discovery of potential therapeutic agents against brain-eating amoeba (Naegleria fowleri)
Source: PLoS One. 2025 Jul 11;20(7):e0327621. doi: 10.1371/journal.pone.0327621 (PMC12250431; doi:10.1371/journal.pone.0327621)
Supplement: S5 Table — (DOCX) [file pone.0327621.s005.docx]

**Table S5. Results of the alignment of *N. fowleri*’s** **α tubulins to *T. gondii*, *P. falciparum* and human α tubulins using BLASTP.** Data shown represents the identity percentages of the pairwise alignment of queries – templates, with the former being the amoeba’s sequences.

| **Organism** | ***N. fowleri***  *α* **5134** | ***N. fowleri***  *α* **7486** | ***N. fowleri***  **Flagellate** *α* |
| --- | --- | --- | --- |
| *T. gondii* | 58.76% | 61.59% | / |
| *P. falciparum* | 59.82% | 62.25 | / |
| Human α Ia | 56.82% | 59.18% | 86.33% |
| Human α Ib | 56.82% | 59.41% | 86.10% |
| Human α Ic | 56.54% | 58.72% | 85.04% |
| Human α IIIc | 57.05% | 59.41% | 86.56% |
| Human α IIId | 57.05% | 59.41% | 86.56% |
| Human α IIIe | 56.36% | 58.73% | 85.42% |
| Human α IVa | 56.10% | 59.38% | 83.26% |
| Human α IVb | 52.22% | 54.68% | 73.06% |
| Human α VIII | 56.49% | 58.96% | 81.32% |
| Human α L3 | 50.45% | 53.26% | 66.14% |

T. = *Toxoplasma*, P. = *Plasmodium*
