# Supplementary material for: Computational discovery of potential therapeutic agents against brain-eating amoeba (Naegleria fowleri)
Source: PLoS One. 2025 Jul 11;20(7):e0327621. doi: 10.1371/journal.pone.0327621 (PMC12250431; doi:10.1371/journal.pone.0327621)
Supplement: S6 Table — (DOCX) [file pone.0327621.s006.docx]

**Table S6. Results of the alignment of *N. fowleri*’s** **β** **tubulins to *T. gondii*, *P. falciparum* and human β** **tubulins using BLASTP.** Data shown represents the identity percentages of the pairwise alignment of queries – templates, with the former being the amoeba’s sequences.

| **Organism** | ***N. fowleri***  β **5966** | ***N. fowleri***  β **7486** | ***N. fowleri***  **Flagellate** β |
| --- | --- | --- | --- |
| *T. gondii* | 63.45% | 64.75% | / |
| *P. falciparum* | 63.45% | 65.21% | / |
| Human β I | 60.70% | 60.61% | 79.68% |
| Human β IIa | 63.81% | 65.35% | 90.23% |
| Human β IIb | 63.57% | 65.58% | 90.70% |
| Human β III | 63.45% | 65.44% | 85.01% |
| Human β IVa | 62.70% | 64.25% | 90.16% |
| Human β IVb | 62.94% | 64.95% | 90.87% |
| Human β V | 62.65% | 64.65% | 90.68% |
| Human β VI | 62.67% | 64.43% | 85.88% |
| Human β VIII | 63.64% | 65.42% | 85.25% |

T. = *Toxoplasma*, P. = *Plasmodium*
