# Supplementary material for: Computational discovery of potential therapeutic agents against brain-eating amoeba (Naegleria fowleri)
Source: PLoS One. 2025 Jul 11;20(7):e0327621. doi: 10.1371/journal.pone.0327621 (PMC12250431; doi:10.1371/journal.pone.0327621)
Supplement: S7 — (DOCX) [file pone.0327621.s007.docx]

**Additional information on the methods**

1. **Homology Modelling**
   1. **Templates selection**

When performing the homology modelling, the ideal similarity value for a template is equal or greater than 60%, since this results in an accuracy of the resulting model that is comparable to experimental structures.

In template selection, sequence similarity is not the only important aspect to consider: resolution, R value and the number of missing residues must be taken into account, since they provide insight on the quality of the template.

The resolution of an experimental structure depends on the method used for its determination. X-ray crystallography generally results in a better resolution than do nuclear magnetic resonance (NMR) or electron microscopy (EM). In X-ray crystallography, a resolution value around 1 Å identifies a high resolution structure, while values above 3 Å identify bad resolution structures. In the latter case, only the overall contour of the molecule is well defined, while the atoms’ positions must be inferred.

The R value measures how well the template’s simulated diffraction pattern differs from the real pattern observed in the laboratory. Good quality structures have an R value around 0.2. A value of 0.6 corresponds to a random placement of the atoms; therefore, the closer the R value is to 0.6, the worse the quality of the structure. Since the R value is the result of a manipulation of the template’s model to adapt it to the experimentally observed diffraction pattern, a less biased evaluation can be made using the R-free, whose typical value is set around 0.26.

Lastly, the presence of missing atoms in the template is an important aspect because the higher their number, the higher the chance of introducing errors in the structure when modelling those atoms. As a general rule of thumb, the presence of missing atoms is of lower importance the more distant they are from the regions of interest in the protein (for example, binding pockets). Missing atoms are usually found in correspondence of loops, highly motile regions whose conformation is difficult to determine. In order to model missing residues, two main methods can be used: *knowledge-based* modelling, that makes use of databases, or *de novo* modelling. The accuracy of this process is dependent on both the number of consecutive missing residues and the method of choice: the knowledge-based is usually more accurate, using the coordinates of homologous database entries, and both are more reliable when the number of residues to model is lower than 10. As an alternative, the presence of missing residues can be handled by using multiple templates for the construction of the homology model, as done in the *segment-based* approach.

- 1. **Preparation of Templates**

Prior to performing the homology modeling, the selected templates were prepared using Chemical Computing Group’s MOE (Molecular Operating Environment) software package. Each template was retrieved from the PDB database by clicking, in MOE’s main window, on *File → Open → RCSB PDB* and then entering each template’s name in the search bar. For each, the number and position of the missing residues was evaluated, and any structure other than the tubulin heterodimer (for example, the tubulin-tyrosine ligase or the stathmin-like structures) was removed as well as the solvent molecules. All ions and ligands were kept, given their relevance to the tubulin structure. MOE’s *Structure Preparation* panel, accessible by clicking on *Compute → Prepare →* *Structure Preparation*, was used to prepare each template. This panel allows easy management of common issues, such as:

- Alternates: residues with alternate locations or ambiguous sequence identities, corrected respectively by using the highest occupancy alternates and deleting other sequence identities.
- Termini: missing atoms in the backbone of the protein’s C- or N- termini, corrected by deleting the residues and capping the terminus.
- Breaks: missing residues inside the chain, corrected by means of loop building.
- Library: missing atoms or inconsistencies between the residues’ names and their structure, corrected by mutating the residues to match the sequence identity;
- Charge: incorrect charges in the system, corrected by applying the charging method of the selected forcefield.

MOE’s Protonate 3D was then used to assign ionization states, optimize hydrogens’ positions in the molecule and calculate partial charges.

To relieve any strain in the structure and solve atom clashes, a minimization step was finally performed with restraints applied to the backbone’s heavy atoms, which allows the relaxation of the system without it being driven away from the starting conformation.

- 1. **Model construction**

Following templates’ preparation, the sequences of *N. fowleri*’s mitotic tubulins were uploaded into MOE as *fasta* files. Since *N. fowleri* presents two *α* and two *β* mitotic tubulin isotypes, two models were built for each of the three selected templates, for a total of six. The isotypes were grouped to form two dimers as follows:

- - - - Dimer 5134-5966, constituted by isotypes *α* 5134 and *β* 5966;
      - Dimer 7486-3784, constituted by isotypes *α* 7486 and *β* 3784.

Both *α* isotypes were included in the models in order to compare the resulting structures and take into account the effects of their different residues in ligands’ interactions with the protein, although the colchicine site resides almost completely on the *β* monomer.

After being uploaded, the *α* and *β* sequences of both the template and the targets were assigned to two separate panels by clicking on *SEQ → Align/Superpose → Set Up Chains*, so that the sequences related to the same subunits were grouped in the same panel. In case the template had more than one dimer, for each monomer we kept only the sequence with the lowest number of missing atoms.

The sequences of the targets and the template were then aligned using the *Align* button of the *Align/Superpose* application. Ligands and ions were ignored in this stage by selecting only the sequences of interest in the sequence alignment window and by pressing the **/i* button in the *Set up chains* application. The goodness of the alignment was evaluated by visual inspection.

MOE’s *Homology Model* application was then started, by clicking on *Protein →* *Homology Model* in the *Sequence editor* window. Each target sequence was assigned to its template upon selection in the *Sequence and Template* drop down menus. Ligands and ions were set to be used as environment, so that their atoms are included in both model construction and energy minimization to prevent atomic clashes or superposition.

In the settings, the number of side chain samples and main chain models were set to 5; all other parameters were left as default. This way, from each template 25 models were built for each dimer. Full refinement as well as optimization of the ionization states were set to be applied to all intermediate models. Since the C- and N-termini are not involved in colchicine binding and are adequately far from its binding site, the related outgap was not modelled. This choice was made also to reduce the likeliness of introducing errors in the structure, since the termini are one of the most common places where the template’s missing atoms are found.

For each dimer-template combination, the resulting 25 models were ranked according to the default scoring method: GB/VI (Generalized Born/Volume Integral), that takes into account Coulomb and Generalized Born interaction energies of both the protein and the atoms set as environment. To select the best model for each dimer, in addition to the GB/VI score, geometrical parameters were considered, namely: the number of atom clashes, bond length outliers, bond angle outliers, torsion outliers and rotamer outliers. A model was considered to be good when having a low number of geometrical issues.

When multiple models were identified as equally good, they were uploaded into MOE and compared in terms of:

- RMSD (root mean square deviation), to evaluate the deviation of each model from the starting template and from the other models under analysis. The reference value used was 0.5 Å, since different experimental determinations of the same protein are documented to differ to this extent.
- Ramachandran plot, to prioritize structures with a low number of *phi-psi* (Φ/Ψ) outliers. This is because remodeling the outliers may introduce errors in the structure, so the fewer the corrections needed the better.

The best models were identified as those providing the best compromise between the GB/VI score and the number of required corrections.

- 1. **Model refinement**

Upon selection of the final model for each dimer-template combination, some corrections were made, if deemed necessary. These included:

1. Loop remodeling of any Ramachandran outlier, using MOE’s *Loop Modeler*

application. Accessible by clicking on *SEQ → Protein → Loop Modeler*, this application allows to remodel the selected residues using either a *knowledge-based* method, that relies on a PDB-derived loops database, or a *de novo* method, which is the suggested choice when the number of residues to remodel is greater than 7. In both cases, the loop to be used as template in the remodeling was chosen by comparing its conformation to the starting loop, so as not to significantly alter the original conformation, and by ensuring that it did not introduce new Ramachandran outliers.

1. A tethered energy minimization, to solve atom clashes.
2. Rotamers correction using MOE’s rotamers library, accessible from the protein

builder application by clicking on *Edit → Build → Protein* in MOE’s main

window.

1. **Computational methods for binding site analysis**

MOE is a useful tool to analyze the physicochemical features of any protein of interest, starting from its three-dimensional model. In our study, this feature was used to evaluate how the interactions landscape and the overall shape of the colchicine site differ from that of the human tubulins, providing complementary information to the alignment with Clustal Omega.

This analysis can be reproduced by uploading a ligand-bound receptor into MOE’s environment and subsequently starting the *Surfaces and maps* application by clicking on *Surface → Surfaces and maps*. In the pop-up window it is possible to select which surface to compute from the *Surface* drop-down menu, and specify the atoms to create the surface for with the *Near* drop-down menu. The *Within* attribute of the *Near* menu allows the user to define the distance in Å to consider as the cut-off for the calculation (i.e., the surface is not computed for atoms beyond the inserted value from those that have been selected).

The Surfaces and Maps application was used to compute the density probability surfaces for both the electrostatic and the non-bonded interactions in the colchicine site, as well as the molecular surface of the pocket.

The molecular surface of the binding pocket gives information about its overall shape and dimensions, according to the Van der Waals radii of the atoms involved. In MOE, it is an approximation of the solvent-excluded surface, also known as the *Connolly surface*. The interaction maps are instead useful to identify the preferred interaction types in the pocket and their location, and are divided into electrostatic maps and non-bonded interactions maps. These maps are computed as density probabilities and depend on the pocket’s conformation. The electrostatic interaction maps are the result of the application of the Poisson-Boltzmann equation (PBE) to the prediction of the electrostatically preferred locations for hydrogen-bond acceptors, hydrogen-bond donors and hydrophobic groups in the binding site. The non-bonded interaction maps identify the preferred locations for hydrophobic and hydrophilic groups from the three-dimensional coordinates of the receptor. Unlike the electrostatic interactions computation, hydrogen atoms and partial charges are not taken into consideration for non-bonded interaction maps.

Given that the study was performed with a rigid receptor, the interpretation of these maps had to be performed with due respect. This was even more true for the colchicine site: since it only appears in the presence of a colchicine site binding ligand, different ligands may result in slightly different conformations of the binding pocket, and focusing on only one of them may introduce a bias in the evaluation of the site’s surfaces. To evaluate the extent to which the colchicine site’s conformation varies when interacting with different ligands, the colchicine-bound model 5EYP, the CA-4-bound model 5LYJ and the TUB092-bound model 5JVD from PDB were aligned and superposed in MOE. Their structures were compared in terms of RMSD and interaction maps in the colchicine site. The result of this analysis can be seen in figures S1 and S2.

**Fig S1. Graphical representation of the comparison of the conformation of PDB entries 5EYP, 5LYJ and 5JVD, with a focus on the colchicine site.** The three structures are color coded according to their relative RMSD. The more intense the green, the lower the RMSD (the more similar the conformation). Yellow, orange and red are used to label regions with RMSD value progressively higher (worse superposition).

**Fig S2. Electrostatic and non-bonded interaction maps in the colchicine site of PDB models 5EYP (cyan), 5LYJ (green) and 5JVD (magenta), computed with MOE.** Subfigures A, C and E depict the electrostatic interaction maps. Subfigures B, D and F depict the contact preferences maps.

The main difference observed in the pocket’s conformation was the position of the side chains of its residues. The backbone conformation was almost identical among the models, except for the loops taking part in the site. This is of no surprise given the intrinsic high mobility of loops, which makes them capable of undergoing conformational changes to adapt to the interacting ligands. This is noticeable by looking at the colchicine site focus in figure S1.

The RMSD between all three structures was 0.661 Å, while the pairwise RMSD was 0.58 Å for 5JVD-5LYJ, 0.63 Å for 5EYP-5JVD and 0.77 Å for 5EYP-5LYJ. This suggests that the different ligands have a little influence on the conformation of the site, especially considering that a 0.5 Å RMSD value is often found among different experimental determinations of the exact same protein. The 5EYP-5LYJ RMSD suggests that greater attention should be given to this aspect when using ligands having significantly different physicochemical properties to the three classes of compounds considered here, which could be the case in the context of ligands derivatization.

The influence of the different ligands is reasonably low on the interaction maps too. Indeed, in figure S2 A, C and E it is possible to observe that the electrostatic interactions profile is very similar among the three models. The central white region, representing an area favorable for hydrophobic interactions, is conserved in all three pockets and is overlapped with shared and corresponding features among the ligands. These are the trimethoxyphenyl (TMP) moiety (on the left side of the ligands’ molecules), the methyl group on the other side of the molecule (in the phenyl ring for CA-4 and TUB092 and in the rightmost seven-membered tropolone ring for colchicine), and the bridging groups for CA-4 and TUB092, all features known to be relevant for these compounds’ activity. Similarly, the disposition of the regions favoring hydrogen-bond interactions around this central hydrophobic core is reasonably maintained. A similar situation can be observed for the non-bonded interactions in figure S2 B, D and F.

Overall, this analysis suggests that it is reasonably safe to focus on only one conformation of the pocket without introducing a significant bias on the interpretation of the maps for both the animal and the amoeba’s tubulins. Of notice, the PDB models compared do not all belong to the same species: model 5EYP belongs to *Ovis aries’* tubulin, while the other two models belong to *Bos taurus’* tubulin. Therefore, the differences observed may also be dependent on the differences in these two species’ tubulin sequences.

1. **Docking simulations**
   1. **Input files description**

The receptors used for the docking simulations were:

- the two homology models of *N. fowleri*‘s mitotic tubulins obtained from PDB entry 5EYP;
- nine homology models of the human tubulins. They derive from PDB entry 5EYP as well, and represent the nine human tubulin *β* isotypes (I, IIa, IIb, III, IVa, IVb, V, VI, VIII) in combination with the human *α*I subunit. Table S6 displays the scores resulting from their validation with UCLA’s SAVES and QMEAN.

The ligands files used correspond to colchicine, thiocolchicine, nocodazole, combretastatin A-4 (CA-4), ombrabulin, TUB092, ST-11, ST-401, compound 5 from Zhou et al.[1] (an oxazole-bridged CA-4 analog), and the best thiazole-bridged CA-4 analog from Ohsumi et al. [2] They were loaded in MOE by using the program’s molecule builder, accessible by clicking on *Edit → Build → Molecule* in the main window, and saved into a database. Colchicine, thiocolchicine, nocodazole, CA-4 and ombrabulin SMILES strings were retrieved from PubChem, and their structures loaded in MOE by using the SMILES box of the molecule builder. TUB092 was isolated from PDB entry 5JVD; the remaining compounds were built manually using the molecule builder, according to the information in the related papers.

- 1. **Files preparation for docking procedure**

MOE did not require any particular preparation of the receptors files, since they were obtained through the homology modelling performed inside its environment, and were already energy-minimized structures.

The ligands preparation was performed following MOE’s guidelines for database preparation. The first step was a washing to correct structural issues, followed by partial charges calculation using the AM1-BCC forcefield, due to its parametrization for small molecules and its compatibility with the Amber10:EHT forcefield, used for the receptors. A final minimization step, to relieve any strain in the structure, concluded the ligands preparation process.

Autodock and Vina require both the receptors and the ligands to be in the PDBQT format. The PDBQT format is similar to the PDB format, adding partial charges, Autodock atom types, torsional and rotational information to a conventional PDB file.

Prior to conversion, all the ions and ligands bound to the tubulin dimers were removed, with the exception of the GTP molecule positioned between the α and β subunits, because of its relevance due to the proximity to the colchicine site.

Upon saving each receptor and ligand of interest as an individual pdb file, the conversion to the pdbqt format was performed using two scripts provided by Autodock-Tools (ADT), *prepare_receptor4.py* and *prepare_ligand4.py*. The former managed the receptors’ preparation – addition of hydrogens, partial charges calculation, non-polar hydrogens and lone-pair merging, heteroatoms removal and Autodock4 atom types definition – and saved the resulting structure in a pdbqt file format. Similarly, *prepare_ligand4.py* managed the ligands’ preparation, saving the result in a separate pdbqt file for each molecule.

- 1. **Search space and docking parameters definition**

In MOE, it is possible to specify the parameters and files to use in the simulation in the docking window, accessible by clicking, in the main window, on *Compute → Dock*. To take into account the presence of the intra-dimer GTP, an atom set containing both the receptor and the GTP molecule was created by selecting the related atoms, and then clicking on *Select → Set create*. This set was named *receptor*, and set as the target of the simulation by selecting the related entry from the *Receptor* drop-down menu of the docking window.

To confine the simulation to the colchicine site, for each receptor the residues composing the target pocket were identified and saved in a new atom set following the same procedure. The identification of the site’s residues in the human tubulin models was performed by selecting the bound colchicine molecule, pressing CTR+L and inserting *rsel ≫ 4.5* in the SVL command line. This allowed to select the atoms within 4.5 Å of the colchicine molecule. The ligand needed to be removed from the selected atoms before the creation of the new set, otherwise the binding site would be seen as already occupied during the simulation, and nothing can be docked inside of it.

An additional step was necessary for the amoeba’s tubulins. Given that the amoeba’s models did not present ligands in the colchicine site because of the way they were built, they were first aligned and superposed on one of the human models, to place the colchicine molecule in the pocket by superposition - i.e., without the need of a docking simulation. The superposition to the human model’s colchicine site was strengthened by performing an additional superposition step, focused on the residues within 4.5 Å of the colchicine molecule, before identifying the final binding site’s residues. The resulting tubulin-colchicine complexes were also saved to be used in the Autodock suite programs, which require a different file preparation.

Finally, to confine the simulation to the site of interest, the corresponding atom set was selected in the *Site* drop-down menu of the docking window. The ligands were instead specified by inserting the corresponding database in the *Ligands* section of the same window; all other parameters were left as default.

In contrast to MOE, Autodock4 and Vina require the compilation of files containing the parameters for the docking simulation, as well as the definition of a box enclosing the target region of the protein. To define the box’s optimal placement and dimension, a software called AMDock was used. The *Center on Hetero* option was selected, that allows us to center the box on a user-specified co-complexed ligand (colchicine in our case) and define the box size accordingly. Given the different sizes of the molecules to dock, the bulkiest ligands were replaced to colchicine and the box’s optimization procedure repeated, to ensure its optimal definition for the whole ligands set. The result was inspected using AMDock’s Pymol viewer.

Upon the definition of the box’s parameters, the box size and its center’s coordinates were stored in a Vina configuration file. For Autodock4, a grid parameter file (*gpf*) was created, containing the box’s information and the ligands’ covering set of atom types. The latter was extracted from the result of the ADT’s script *prepare ligand4.py* using the option -d. Autogrid4 was then used to calculate the required set of atom maps, and finally a docking parameter file (*dpf*) for each ligand-receptor pair was generated with the ADT script *prepare dpf4.py*.

- 1. **Consensus Docking Approach**

Currently, approximately 60 docking programs are accessible either for commercial use or academic purposes. These vary primarily in how they represent the receptor, the flexibility they allow in the molecules, the search methods they employ, and the scoring functions they utilize. This variety can lead to discrepancies in the results when different programs are used to simulate the same ligand-receptor complex.

To mitigate the limitations inherent in individual docking programs and enhance result reliability, a consensus docking method was employed in the present study involving MOE, Autodock4 and Vina. This method involves using multiple docking programs to simulate the same interaction independently and compare their outcomes.

- 1. **Benchmarking Docking Programs**

In consensus docking, it is crucial to evaluate how well each program can predict the ligands’ native pose — the experimentally determined location and orientation of a compound when interacting with its target. This evaluation, known as benchmarking, can only be conducted when the native poses are known.

To conduct the benchmarking process in our experiments, the known native poses of Colchicine, Combretastatin A-4, and the Chalconoid TUB092 were referenced from PDB entries 5EYP, 5LYJ, and 5JVD, respectively. Each of the three ligands was docked onto each human tubulin dimer. The top poses were saved as mol2 files and compared, using DockRMSD, to the native pose in the related PDB structure. If the best pose’s RMSD exceeded 2 Å, the second-best pose was analyzed. In case of a negative outcome, a new docking run was performed. If after 10 runs no match was found, the threshold was adjusted to 2.5 Å and the process repeated. If still no consensus emerged, the software was deemed unlikely to produce a reliable prediction for that ligand-protein combination. Programs failing to accurately predict the native pose for at least half of the receptors were discarded.

- 1. **Consensus Protocol for the Chosen Ligands**

A different consensus protocol was used to generate and evaluate the docking results for the colchicine analogs chosen for in silico testing. This consensus protocol focused on an initial evaluation of the internal consistency of each software in reproducing the top poses for the same ligand-receptor complex, followed by the assessment of the consensus among the three docking programs.

For each ligand-receptor pair, a minimum of 3 runs of docking simulations was carried out with each docking software. For each program, the top pose of each run was extracted and compared with the other two in terms of binding energy score. If all three scores were within 0.1 kcal/mol from each other, the poses were loaded into ADT viewer for AutoDock4 and Vina or MOE’s viewer otherwise and inspected to ensure pose similarity and location inside the expected binding region. If the scores did not point to an internal consistency, a novel run was performed, and the analysis repeated.

Once assessed the intra-consistency for each software, the top poses for each ligand-receptor pair were compared among programs, looking for an agreement in terms of placement inside the binding region, binding pose and binding energy score.

The selection criteria for the best poses ensured that the ligands not only occupied the colchicine pocket fully but also aligned their functionally critical features with analogous regions within the experimentally determined PDB structures of colchicine, CA-4, and TUB092 sourced from the homology templates. This aimed at emphasising the alignment of similar functional groups within the binding pocket, while also accounting for the anticipated sequence substitutions in the amoeba's site.

**References**

1. Zhou J, Jin J, Zhang Y, Yin Y, Chen X, Xu B. Synthesis and antiproliferative evaluation of novel benzoimidazole-contained oxazole-bridged analogs of combretastatin A-4. European Journal of Medicinal Chemistry. 2013;68: 222–232. doi:10.1016/j.ejmech.2013.08.006

2. Ohsumi K, Hatanaka T, Fujita K, Nakagawa R, Fukuda Y, Nihei Y, et al. Syntheses and antitumor activity of cis-restricted combretastatins: 5-membered heterocyclic analogues. Bioorganic &amp; Medicinal Chemistry Letters. 1998;8: 3153–3158. doi:10.1016/s0960-894x(98)00579-4
