# Supplementary figures and images for: Computational discovery of potential therapeutic agents against brain-eating amoeba (Naegleria fowleri)
Source: PLoS One. 2025 Jul 11;20(7):e0327621. doi: 10.1371/journal.pone.0327621 (PMC12250431; doi:10.1371/journal.pone.0327621)

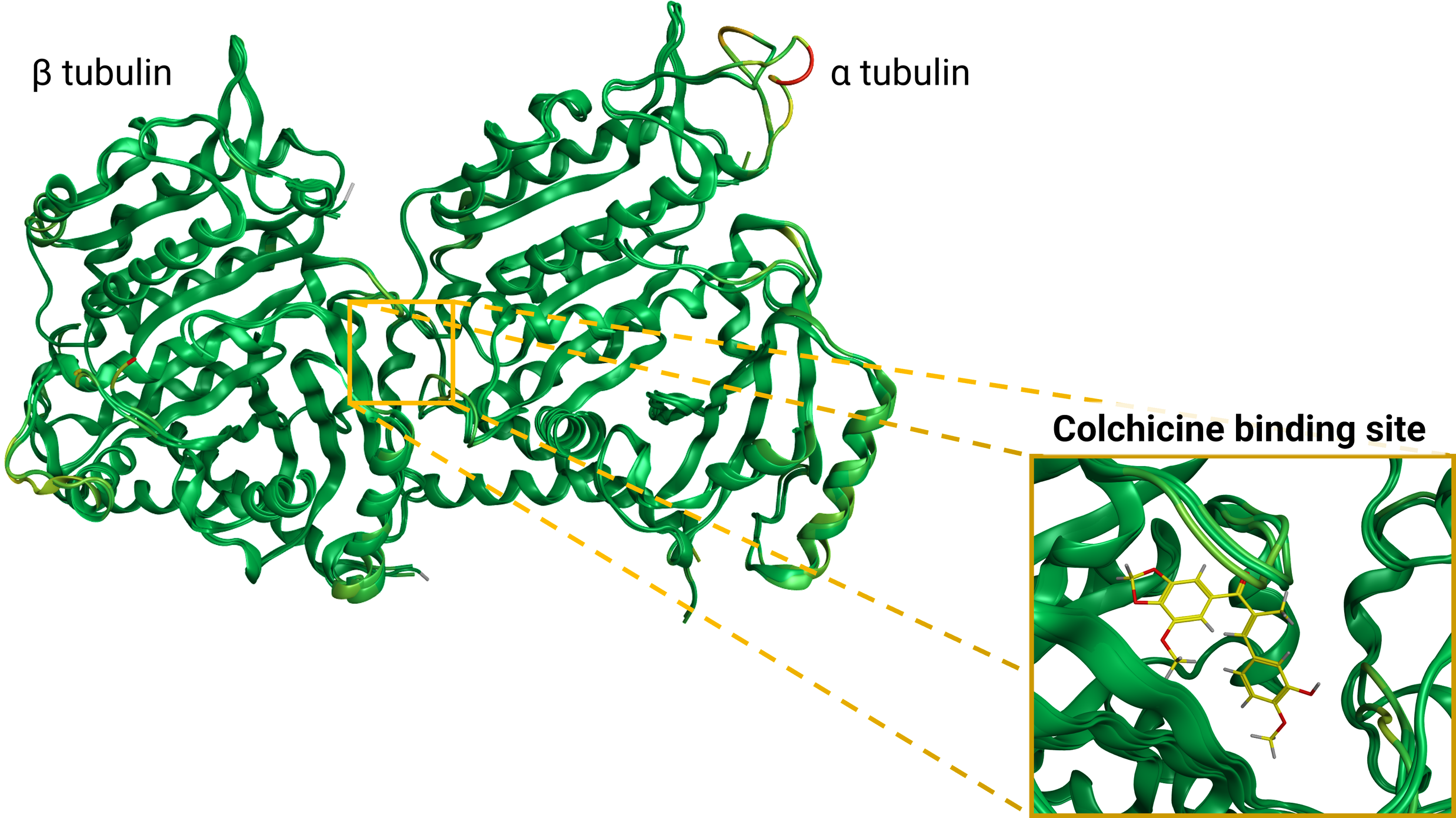

Supplement: S1 Fig — (TIF) [file pone.0327621.s008.tif]

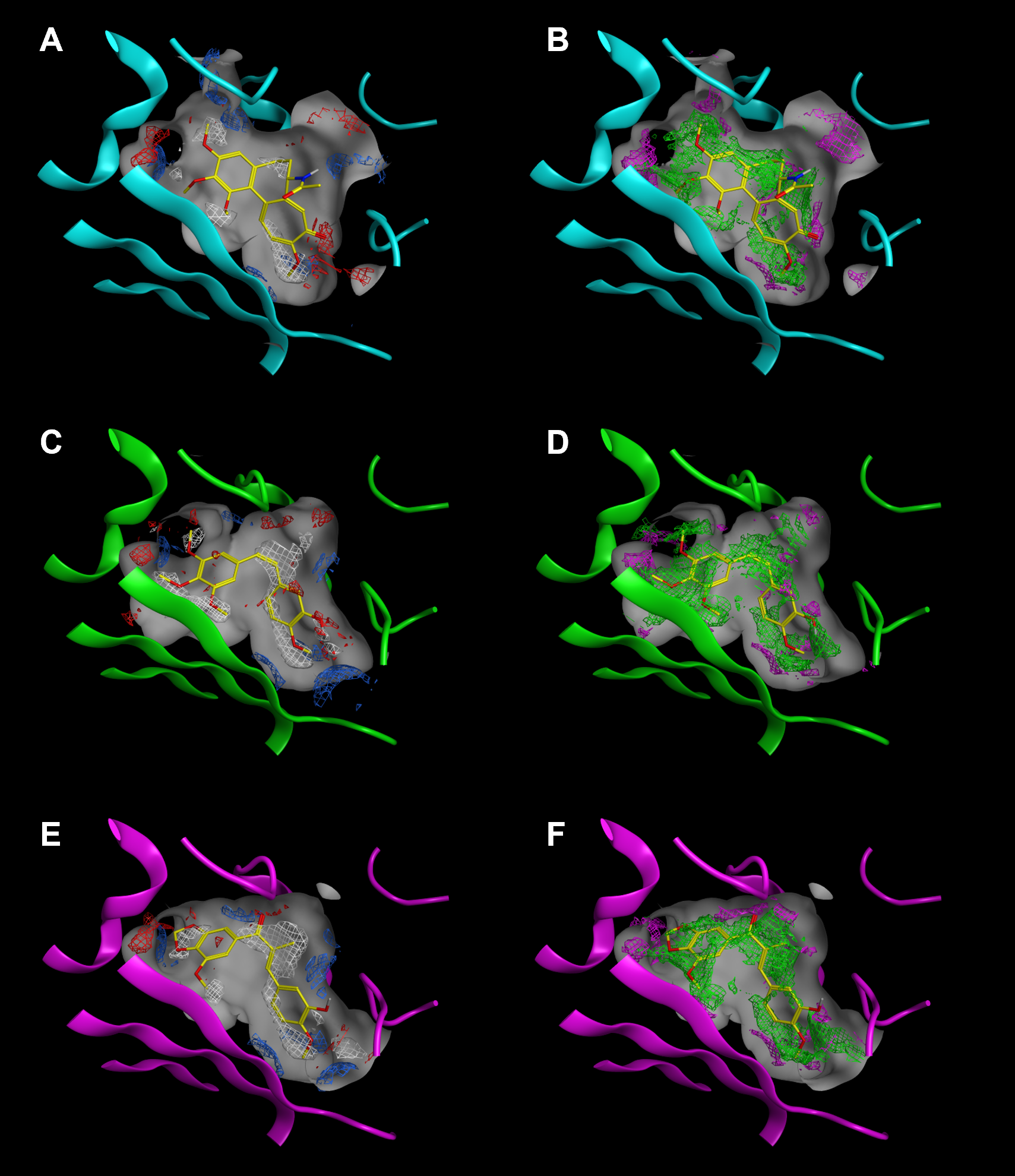

Supplement: S2 Fig — (TIF) [file pone.0327621.s009.tif]
